# FOXO1-mediated nuclear sequestration of STAT3 and AKT1 triggers FOXO3-dependent autophagic death in hypoxic granulosa cells

**DOI:** 10.7150/ijbs.101309

**Published:** 2024-11-04

**Authors:** Chengyu Li, Gang Wu, Caibo Ning, Zhaojun Liu, Jingli Tao, Xiumei Lu, Ming Shen, Honglin Liu

**Affiliations:** College of Animal Science and Technology, Nanjing Agricultural University, Nanjing 210095, China.

**Keywords:** FOXO1, FOXO3, STAT3, AKT1, Autophagic death, Hypoxia, Granulosa cells

## Abstract

FOXO proteins, especially FOXO1 and FOXO3, are recognized for their roles in controlling apoptosis and autophagy. Both apoptosis and autophagy have been induced in granulosa cells (GCs) by hypoxic conditions in ovarian follicles; however, the exact contribution of FOXO proteins and autophagy to the regulation of GCs apoptosis under hypoxia remains unclear. In this investigation of porcine GCs, we reveal that FOXO1 promotes apoptosis in response to hypoxia through FOXO3-dependent autophagy. We describe how mechanistically, FOXO1 forms a complex with the transcription factor STAT3 during hypoxia. Guided by FOXO1, this complex undergoes nuclear translocation and effectively attaches to the STAT3-responsive element (SRE) located in the FOXO3 promoter region, thereby enhancing the transcriptional expression of FOXO3. Simultaneously, FOXO1 associates with AKT1, thus facilitating its nuclear entry and subsequently reducing the Ser253 phosphorylation of FOXO3, leading to FOXO3 detachment from 14-3-3 and promoting FOXO3 translocation into the nucleus. FOXO3 subsequently stimulates the upregulation of ATG3, ultimately initiating autophagy and autophagy-dependent apoptosis. Our results suggest that hypoxia acts through FOXO1 to induce autophagic death in porcine GCs by promoting the expression and nuclear import of FOXO3.

## Introduction

The impact of hypoxia on the functionality of the ovaries has become increasingly recognized. Among the discoveries, hypoxia has been found to deplete follicular reserves in spiny mice, hinder follicular development in hamsters, and induce the production of reactive oxygen species (ROS) in and accelerate the aging process of human granulosa cells (GCs) 1. Although cells initially deploy adaptive and survival strategies in response to oxygen deprivation, prolonged hypoxia can activate different cell death mechanisms, including autophagy, apoptosis, necrosis, and necroptosis, which are influenced by the intensity and length of hypoxic conditions 2-4. Autophagy, a well-conserved intracellular degradation process, helps maintain cellular homeostasis during stress but can result in autophagic cell death if excessively activated 5. Current research indicates that autophagy plays a role in modulating the process of follicular atresia 1, which is characterized by the extensive apoptosis of GCs 6. However, the precise interplay among autophagy, apoptosis, and hypoxia in ovarian GCs remains largely undefined.

Forkhead box proteins (FOXOs) represent a group of transcription factors conserved across evolution in organisms ranging from yeast to humans. This family is composed of FOXO1, FOXO3, FOXO4, and FOXO6 in mammals. The roles of FOXO proteins are regulated by their post-translational modifications and changes in subcellular localization, which occur in response to various environmental signals 7. For example, upon stimulation by growth factors and insulin, FOXO1, FOXO3, and FOXO4 experience phosphorylation at particular sites, including Thr32, Ser253, and Ser315 on human FOXO3, in an AKT1-dependent manner. AKT1-mediated phosphorylation triggers the cytoplasmic localization of FOXO proteins, which in turn inhibits their transcriptional activity 7. The class I PI3K enzyme serves as a vital upstream regulator of AKT1, initiating the activation of phosphoinositide-dependent kinase-1 (PDK1), which subsequently phosphorylates and activates AKT1 8.

FOXOs serve as transcriptional activators or repressors by interacting with the FOXO-recognized element (FRE) located in the promoters of target genes. Our previous research showed that FOXO1 directly binds to the FRE site located in its own promoter region 9. Aligning with our findings, prior studies have suggested that FOXO3 exhibits the capacity to bind and activate both the FOXO1 and FOXO3 promoters in various cell types 10, 11. Given that all of the members of the FOXO family share the FRE consensus DNA-binding sequence 7, this form of positive feedback mechanism appears to be a common regulatory feature within the FOXO genes.

It is evident that FOXO proteins can influence transcriptional responses independently of direct DNA binding. Studies examining overexpression have indicated that a FOXO mutant lacking DNA binding activity can still regulate target gene expression 12. FOXOs occasionally form complexes with other factors to regulate downstream transcriptional activity 13. In addition to their roles in transcriptional regulation, FOXOs may also influence the activity of other proteins through protein-protein interactions. As reported, when FOXO1 binds to β-catenin, β-catenin is unavailable to bind to T-cell factor, thereby reducing T-cell factor activity and inhibiting β-catenin-mediated bone formation 14.

Among the FOXO family members, FOXO3 has been the most extensively studied member, found to orchestrate various cellular and physiological processes including proliferation, differentiation, apoptosis, metabolism, stress response, and longevity 7. Prior work has established that FOXO3 plays a crucial role in regulating follicle growth and atresia by promoting the apoptosis of GCs and oocytes in mammalian ovaries 15. FOXO3 is also known to be activated in response to hypoxic stress 16, and emerging evidence implicates FOXO3 in the induction of autophagy 17. However, the role of FOXO3 and autophagy in the modulation of GC apoptosis under hypoxia remains unclear.

We report that FOXO1 mediates hypoxia-induced autophagic death by promoting the expression and nuclear transport of FOXO3 in ovarian GCs, 293T cells, and NIH/3T3 cells. Intriguingly, FOXO1 activates FOXO3 expression without direct DNA binding under hypoxia exposure. Instead, FOXO1 forms a complex with the transcription factor STAT3, translocates into the nucleus, and binds with the STAT3-responsive element (SRE) within the *FOXO3* promoter, leading to the transactivation of *FOXO3*. Concurrently, FOXO1 associates with AKT1 and guides it into the nucleus. This interaction reduces phosphorylation of FOXO3 at Ser253, resulting in its dissociation from 14-3-3 and subsequent nuclear translocation. FOXO3, in turn, upregulates ATG3 protein levels, fostering autophagy and autophagy-dependent apoptosis under hypoxia. Our findings offer a mechanism wherein FOXO1 orchestrates the coordinated regulation of FOXO3 to modulate autophagy in the context of hypoxia.

## Materials and Methods

### Reagents and antibodies

The 3-methyladenine (3-MA; S2767), chloroquine (CQ; S6999), and SP600125 (S1460) were purchased from Selleck Chemicals (Houston, TX, USA). IGF-I (10598-HNAY1) was obtained from Sino Biological (Beijing, China). AKT1, FOXO1, and FOXO3-related plasmids were purchased from KeyGEN BioTECH (Nanjing, China). Antibodies against FOXO1 (2880), FOXO3 (2497), p-FOXO3 (Ser253; 9466), cleaved caspase-3 (9664), 14-3-3 (8312), STAT3 (9139), p-STAT3 (Tyr705; 9145), AKT1 (9272), p-JNK1/2 (Thr183/Tyr185) (4668), JNK1/2 (9252), ATG3 (3415), ATG5 (9980), Beclin1 (3738), ATG7 (8558), SQSTM1 (88588), α-tubulin (2125), MAP1LC3B (3868), and FLAG-tag (14793) were purchased from Cell Signaling Technology (Danvers, MA, USA).

### Cell culturing and experimental interventions

GCs from mature Duroc, Yorkshire, and Landrace sows were isolated from ovarian follicles obtained at a local abattoir using a 10 mL syringe. The cells were then cultured in Dulbecco's Modified Eagle Medium (DMEM)/F-12 medium (Life Technologies, Carlsbad, CA, USA) containing 10% fetal bovine serum (FBS; Sigma-Aldrich, St. Louis, MO, USA) and 100 U/mL penicillin/streptomycin (Gibco, Waltham, MA, USA) at 37°C in a 5% CO_2_ humidified incubator. NIH/3T3 or 293T cells were maintained in DMEM (Gibco) enriched with 10% FBS and 100 U/mL penicillin/streptomycin (Gibco) under conditions of 37°C and 5% CO_2_ in a humidified incubator.

For hypoxic exposure, cells were placed in a modulator incubator in an atmosphere of 94% N_2_, 5% CO_2_, and 1% O_2_, with normoxic conditions defined as 21% O_2_. For RNA interference, cells were transfected with *FOXO3-*siRNA, *FOXO1-*siRNA,* ATG3-*siRNA, or scrambled control siRNA for 12 h, followed by overexpression with FOXO1-related vectors under normoxia or hypoxia for another 12 h. In certain experiments, cells were incubated under normoxic (21% O_2_) or hypoxic (1% O_2_) conditions, with or without IGF-I, for the specified durations. For inhibitor studies, cells were pre-treated with 3-MA (an autophagy inhibitor) or SP600125 (a JNK1/2 inhibitor) for 2 h before exposure to hypoxia.

### Gene silencing via RNA interference (RNAi)

siRNAs targeting *FOXO3*, *FOXO1*, and *ATG3* along with scrambled control siRNAs (refer to Supplementary Table S1 for sequences) were sourced from GenePharma (Shanghai, China). siRNA transfection was conducted using Lipofectamine 3000 (Invitrogen, Waltham, MA, USA, L3000015) following the protocol of the manufacturer.

### Confocal imaging of autophagosome formation

GCs plated on coverslips were transfected with the GFP-LC3 expression plasmid (Addgene, Watertown, MA, USA, 22418) using Lipofectamine 3000. After 24 h, cells were treated with or without 3-MA and IGF-I for the specified duration, and then incubated under normoxic (21% O_2_) or hypoxic (1% O_2_) conditions for 12 h. GFP-LC3 puncta formation was observed using a Zeiss LSM 710 META confocal microscope (Carl Zeiss, Jena, Germany).

### Isolation of nuclear and cytoplasmic protein fractions

Cytosolic and nuclear extracts were prepared using the NE-PER Nuclear and Cytoplasmic Extraction Reagents (Thermo Fisher Scientific, Waltham, MA, USA, 78833). Experimental protocols were followed according to the guidelines of the manufacturer. Nuclear and cytosolic extracts were prepared for subsequent western blotting or immunoprecipitation (IP) analyses.

### Immunoprecipitation and western blot analysis

For IP assay, treated GCs were rinsed with phosphate-buffered saline (PBS; Gibco) and lysed on ice using IP lysis buffer (Pierce, Waltham, MA, USA, 26149) supplemented with protease inhibitor cocktail (Roche, Basel, Switzerland 04693132001). Whole-cell lysates (WCL) were subjected to immunoprecipitation with anti-FOXO1, anti-FLAG-tag, or anti-14-3-3 antibodies. In each IP reaction, 5 μl of antibody was added to 500 μl of cell lysate to achieve a final concentration of 20 ng/μl and the mixture incubated at 4°C overnight. After 25 μl of Protein A/G Magnetic Beads (Thermo Fisher Scientific, 88802) was added, incubation was continued for another hour at 4°C. The beads were then collected magnetically before the supernatant was discarded, and the immunoprecipitates were washed with 1× cell lysis buffer, magnetized again to remove the supernatant, and eluted with sodium dodecyl sulfate (SDS) loading buffer (SunShineBio, Nanjing, China). The samples were then processed for immunoblotting with specific antibodies.

For immunoblotting, cells were lysed in ice-cold RIPA buffer (Beyotime, Shanghai, China, P0013B) containing a protease inhibitor cocktail (Roche, 04693132001), and protein concentration was measured using a BCA Protein Assay Kit (Beyotime, P0012). Protein extracts were denatured with SDS (Biosharp, Hefei city, China; BL502A) by boiling for 15 min, separated by electrophoresis on a 4% to 20% Sure PAGE gel (Genscript, Nanjing, China), and transferred to polyvinylidene fluoride (PVDF) membranes (Millipore, Bedford, MA, USA) via electroblotting. Membranes were blocked with 5% bovine serum albumin in Tris-buffered saline (TBST; Solarbio, Shanghai, China) for 1 h to prevent nonspecific binding. Primary antibodies (1:1000) were applied in blocking buffer overnight at 4°C, followed by incubation with horseradish peroxidase (HRP)-conjugated secondary antibodies (1:2000) for 1 h at room temperature. Detection of protein bands was performed using a WesternBright ECL HRP Substrate Kit (Advansta, San Jose, CA, USA) per the instructions from the manufacturer. Relative protein expression was normalized against *TUBA1A* used as the loading control.

### Quantitative real-time polymerase chain reaction (qRT-PCR)

Total RNA was isolated using TRIzol reagent (Invitrogen, Waltham, MA, USA) and converted to cDNA using the PrimeScriptTM RT Master Mix (Takara Bio, Shiga, Japan) according to the instructions from the manufacturer. Quantitative real-time polymerase chain reaction (qRT-PCR) was conducted with AceQ qPCR SYBR Green Master Mix (Vazyme, Nanjing, China) and gene-specific primers (primer sequences are listed in Supplementary Table S2) on an ABI QuantStudio5 system (Applied Biosystems, Waltham, MA, USA). Amplification specificity was verified by analyzing the melting curves. Expression levels were normalized to the housekeeping gene *TUBA1A*.

### Molecular docking of FOXO1 and STAT3 proteins

The protein-protein docking study was conducted using AlphaFold2-predicted structure files of porcine FOXO1 and STAT3 downloaded from the Uniprot database (https://www.uniprot.org/). Molecular docking was performed using ClusPro software (version 2.0) to dock the FOXO1 and STAT3 protein structures. After that, the results were visualized, and the binding pose with the lowest docking energy, which is indicative of the highest binding affinity between the two proteins, was selected as the structure of the two-protein binding complex. The visualization was conducted using Pymol software, and key amino acid residues located on the docking interaction surface were labeled to highlight critical interaction regions.

### Chromatin immunoprecipitation (ChIP) assay

Chromatin immunoprecipitation (ChIP) assays were performed according to the instructions from the manufacturer using a Pierce Agarose ChIP Kit (Thermo Fisher Scientific, 26156). Briefly, cells were cross-linked with 1% formaldehyde for 10 min at room temperature, and the reaction was halted by adding glycine to a final concentration of 0.125 M. After washing with cold PBS containing protease inhibitors, cells were lysed in SDS lysis buffer (1% SDS, 10 mM of EDTA, 50 mM of Tris, pH 8.1). Chromatin was enzymatically sheared using micrococcal nuclease, producing DNA fragments between 100 and 700 bp. For each ChIP experiment, 10% of the chromatin was reserved as input, and the remainder was immunoprecipitated with antibodies against STAT3, RNA polymerase II (positive control), or rabbit IgG (negative control) at 4°C overnight. Following protein digestion with proteinase K, ChIP-purified DNA was extracted and used as a template for qRT-PCR with primers listed in Supplementary Table S3. PCR products were analyzed by electrophoresis on a 2% agarose gel. The relative amount of immunoprecipitated DNA was quantified as a percentage of input chromatin.

### Luciferase reporter assay

The *FOXO3* promoter (2000 bp, 5' UTR of FOXO3) was amplified by PCR from mouse genomic DNA and inserted into the pGL3-Basic vector (Promega, Fitchburg, WI, USA) at the KpnI and XhoI restriction sites (Takara Bio), generating the pGL3-FOXO3 (WT) construct (primer sequences are shown in Supplementary Table S4). Two SRE motifs (GTTCAGGGAAG and CAAGGGCCTTA) within the *FOXO3* promoter were identified by using the JASPAR database (http://jaspar.genereg.net/). Mutations were introduced into these SRE sites using a Mut Express II Fast Mutagenesis Kit (Vazyme) to create pGL3-FOXO3 (mutation I) and pGL3-FOXO3 (mutation II). GCs were transfected with the plasmids using Lipofectamine 3000 (Invitrogen). Every treatment involved co-transfection of three plasmids, namely, (1) 1.6 μg of the expression vector pCMV5-FLAG-FOXO1 or other FOXO1-related constructs; (2) 1.6 μg of the reporter construct pGL3-FOXO1 (WT) or pGL3-FOXO1 (mutation); and (3) 32 ng of the control reporter pRL-TK (Promega). Following 24 h of transfection, luciferase activity was measured using a Dual-luciferase Reporter Assay System (Promega) and a Modulus Microplate Luminometer (Turner BioSystems, Sunnyvale, CA, USA). The data are presented as firefly luciferase activity normalized to *Renilla* activity.

### TUNEL staining

TUNEL staining was performed on GCs using a Cell Apoptosis Detection Kit (Beyotime, C1091) according to the instructions from the manufacturer. In brief, GCs were cultured on coverslips in 12-well plates. After the respective treatments, the cells were washed with PBS, fixed with 4% paraformaldehyde for 30 min at room temperature, and rinsed with PBS. Membrane permeabilization was conducted using ice-cold 1% Triton X-100 for 5 min. Cells were treated with 3% H_2_O_2_, followed by the addition of 50 μL of TdT enzyme reaction mix to each coverslip in order to block endogenous peroxidase activity. Cells were incubated at 37°C in the dark for 1 h and then further incubated with streptavidin-HRP working solution for another 1 h at 37°C in the dark. After PBS washe 3 times, nuclei were counterstained with hematoxylin for 3 min, and DAB solution was used for 3 min at room temperature to visualize the apoptotic signals. Apoptosis in GCs was observed using a 40× objective lens under a light microscope (Olympus Corporation, Tokyo, Japan).

### Data analysis and statistical evaluation

Statistical analysis was conducted using SPSS version 20.0 (IBM, Armonk, NY, USA). Data are presented as the mean ± standard error of the mean (SEM). Experiments were performed in triplicate. Group differences were evaluated by performing one-way analysis of variance (ANOVA) followed by least significant difference (LSD) post hoc analysis. A p-value of less than 0.05 was deemed to be statistically significant. *P < 0.05, **P < 0.01, ***P < 0.001; NS, not significant (P > 0.05).

## Results

### Hypoxia promotes apoptosis by activating autophagy

**Figure 1:**
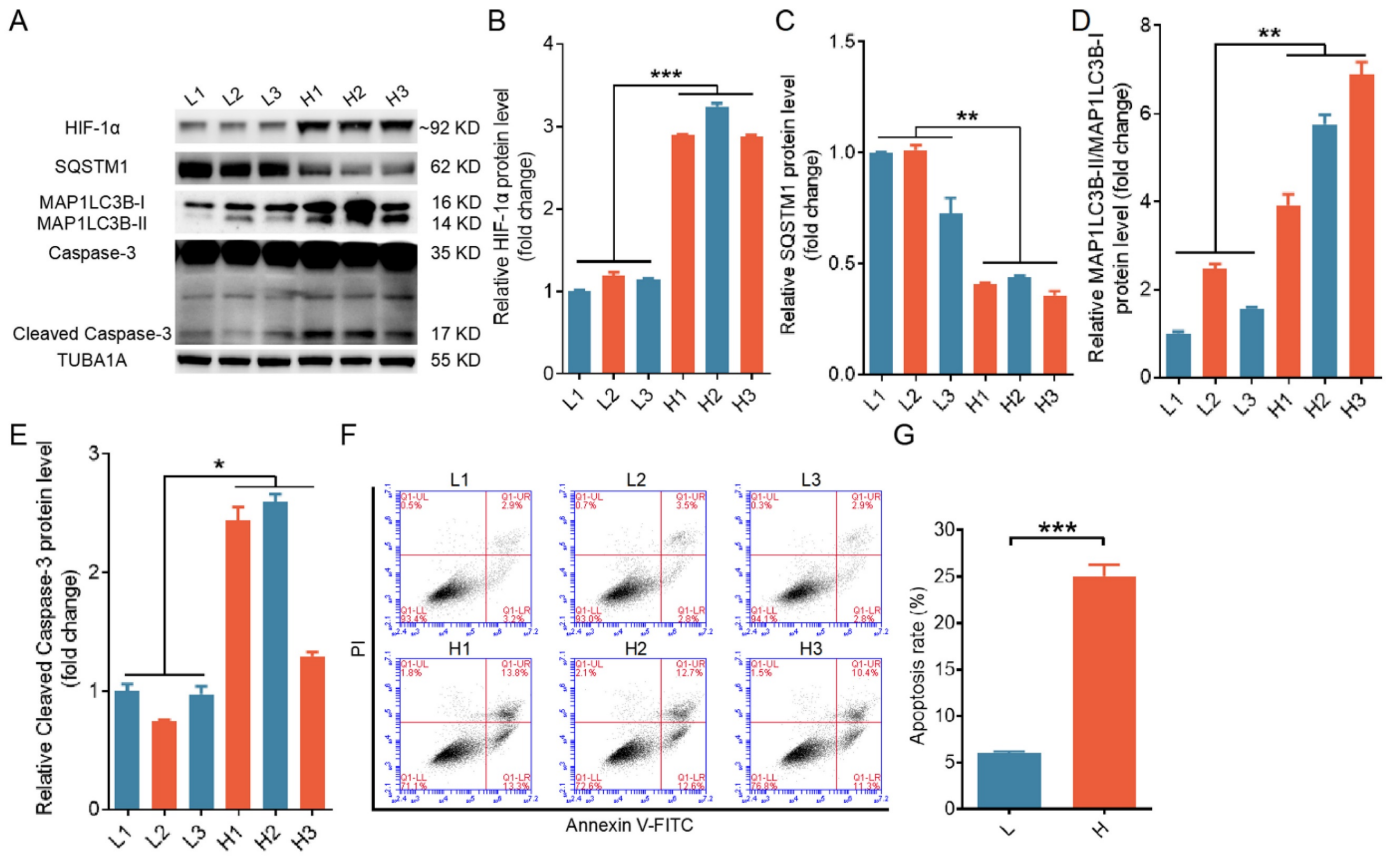
Elevated levels of both autophagy and apoptosis were detected in ovarian granulosa cells (GCs) experiencing hypoxia. Based on HIF-1α protein levels, GCs collected from porcine ovarian follicles were classified into groups with a high level of hypoxia **(H)** and a low level of hypoxia **(L)**. **(A-E)** The protein levels of HIF-1α, SQSTM1, MAP1LC3B, and cleaved caspase-3 in GCs were assessed by western blot and quantified with densitometric analysis using ImageJ software. **(F and G)** The apoptosis rates of GCs were determined by flow cytometry **(F)**, and the percentage of apoptotic cells is shown **(G)**.

To explore the interplay among autophagy, apoptosis, and hypoxia in ovarian GCs, we classified GCs retrieved from porcine antral follicles measuring approximately 4 mm in diameter into two groups, one with relatively high hypoxia and the other with relatively low hypoxia, based on the accumulation of HIF-1α within the cells. Porcine GCs under more hypoxic conditions displayed higher expression levels of MAP1LC3B-II and cleaved caspase-3, along with increased apoptosis rates, as illustrated in Fig. 1A to G.

**Figure 2:**
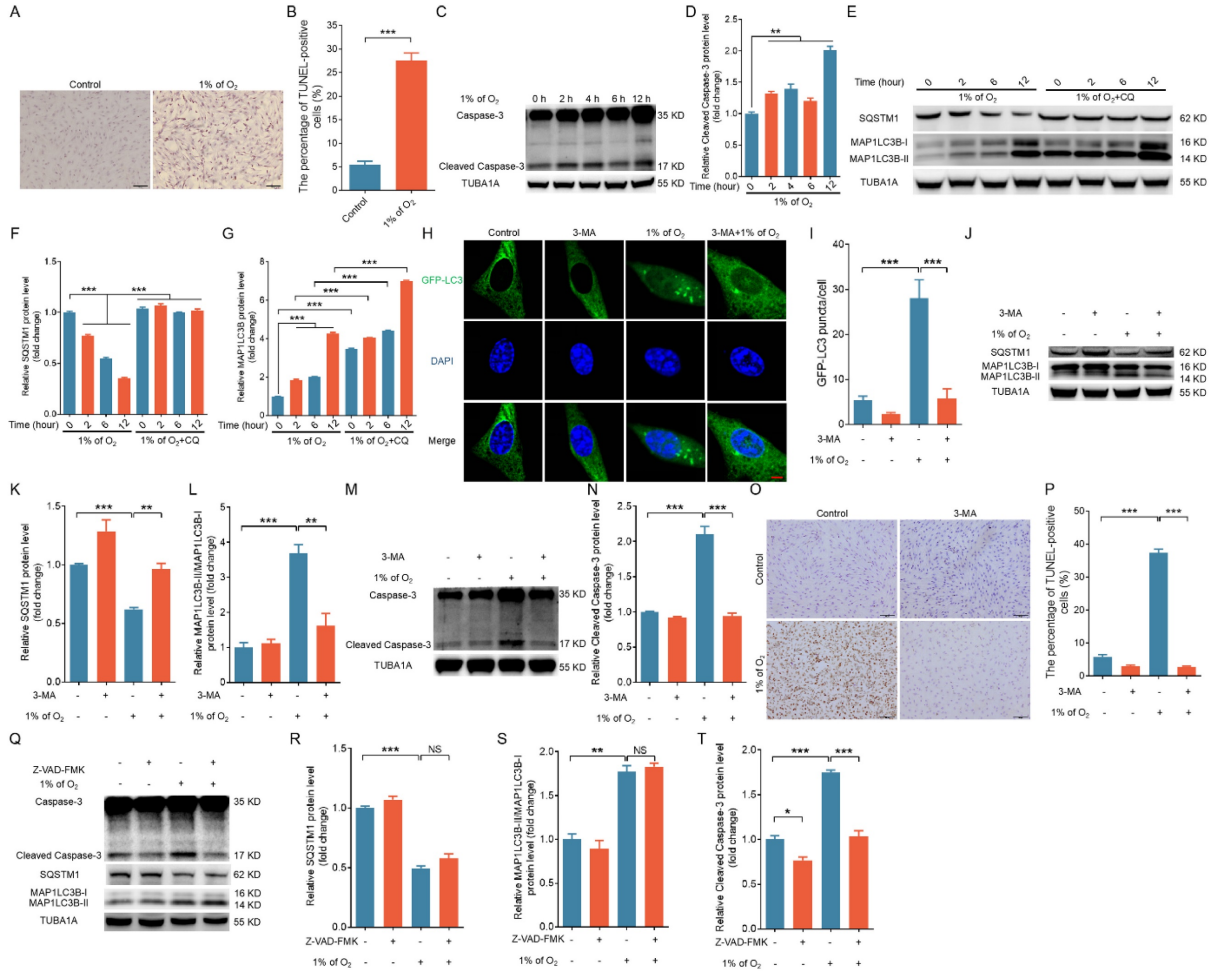
Hypoxia-triggered apoptosis relies on the activation of autophagy. **(A)** Porcine GCs were exposed to either normal (21% O_2_) or low (1% O_2_) oxygen levels for 24 h, and cell apoptosis was evaluated through TUNEL staining. **(B)** The proportion of cells positive for TUNEL staining is shown. **(C and D)** Porcine GCs were incubated under low oxygen conditions (1% O_2_) for 0, 2, 4, 6, or 12 h. Cleaved caspase-3 protein expression was evaluated by western blot **(C)** and quantified through densitometry with ImageJ software **(D)**. **(E-G)** Porcine GCs were subjected to pretreatment either in the presence or absence of 50 μM of chloroquine (CQ) and then cultured under hypoxia (1% O_2_) for 0, 2, 4, 6, or 12 h. The protein levels of SQSTM1 and MAP1LC3B were determined by western blot **(E)** and quantified through densitometry with ImageJ software **(F and G)**. **(H-P)** After being transfected with GFP-LC3 for 12 h, porcine GCs were exposed to either normal (21% O_2_) or low (1% O_2_) oxygen conditions for another 12 h in the presence or absence of 5 mM 3-MA. The autophagic puncta were visualized using laser confocal-scanning microscopy **(H)**. The data were quantitatively analyzed **(I)**. The protein levels of SQSTM1, MAP1LC3B **(J)**, and cleaved caspase-3 **(M)** were determined by western blot analysis and quantified with densitometric analysis using ImageJ 1.42q software **(K, L, and N)**. The apoptosis rate of GCs was assessed using TUNEL staining **(O)**, and the percentage of TUNEL-positive cells was quantified **(P)**. **(Q-T)** Porcine GCs were first exposed to 50 μM of Z-VAD-FMK, a universal inhibitor of caspases, for 2 h. Subsequently, the cells were cultured in either normal (21% O_2_) or low (1% O_2_) oxygen environments for 12 h. After incubation, the expression levels of cleaved caspase-3, SQSTM1, and MAP1LC3B proteins were determined through western blot **(Q)**, and the intensity of the protein bands was analyzed by densitometry with ImageJ software **(R-T)**.

We maintained primary porcine GCs in a hypoxic environment (1% O_2_) as previously described in order to simulate the hypoxic conditions within ovarian follicles 18. Using TUNEL staining, we observed that the apoptosis rate was significantly increased in hypoxia-treated GCs (Fig. 2A and B). Western blot analysis revealed that exposure to hypoxia led to a time-dependent increase in cleaved caspase-3 and MAP1LC3B-II levels, whereas the abundance of SQSTM1 protein decreased (Fig. 2C-G). We obtained similar results for 293T cells and NIH/3T3 cells (Fig. S1A-D), implicating the activation of both apoptosis and autophagy upon hypoxic stimulation.

We examined autophagic flux by tracking SQSTM1 and MAP1LC3 in the presence of CQ, an inhibitor of lysosomal protease to more accurately assess hypoxia-induced autophagy activation (Fig. 2E-G, Fig. S1B and D). We observed the accumulation of MAP1LC3-II in hypoxic cells treated with CQ, thus suggesting that autophagic flux was elevated in response to hypoxia exposure. We employed the autophagy inhibitor 3-MA to suppress cellular autophagy in porcine GCs, 293T cells, and NIH/3T3 cells to elucidate the relationship between autophagy and apoptosis under hypoxia (Fig. 2H-L, Fig. S1E, F). We observed that the inhibition of autophagy completely counteracted hypoxia-induced apoptosis in GCs, 293T cells, and NIH/3T3 cells (Fig. 2M-P and Fig. S1E, F). However, treatment with the caspase inhibitor Z-VAD-FMK did not affect the autophagic activity of hypoxia-treated cells (Fig. 2Q-T). These data indicate that hypoxia-induced apoptosis is dependent on the activation of autophagy; in other words, cells undergo autophagic death in response to hypoxia.

### Activation of the JNK1/2-FOXO1 pathway is required for hypoxia-induced autophagic death

Our prior investigation established that hypoxia induces apoptosis in porcine GCs through JNK1/2-mediated nuclear translocation of FOXO1 19. Concurrent research suggests a correlation between JNK1/2 activation and the occurrence of cellular autophagy under hypoxic conditions 20. Thus, we aimed to ascertain whether hypoxia-induced autophagic cell death in porcine GCs depends on the activation of the JNK1/2-FOXO1 signaling pathway. Hypoxia significantly increased the level of JNK1/2 phosphorylated at Thr183/Tyr185 (Fig. S2A), as depicted in Fig. S2. Treatment with the JNK1/2 inhibitor SP600125 strongly inhibited hypoxia-induced nuclear shifting of FOXO1 (Fig. S2B and Fig. S3A, C), as well as the expression of cleaved caspase-3 and MAP1LC3B-II (Fig. S2C and Fig. S3B, D) in porcine GCs, 293T cells, and NIH/3T3 cells. The elevated levels of cleaved caspase-3 and MAP1LC3B-II upon hypoxic stimulation were also consistently diminished following the knockdown of FOXO1 (Fig. S2D). These findings suggest that the activation of JNK1/2-FOXO1 signaling is a critical step for hypoxia-induced autophagic death.

### Upregulated expression and nuclear translocation of FOXO3 under hypoxia contribute to autophagy/apoptosis

**Figure 3:**
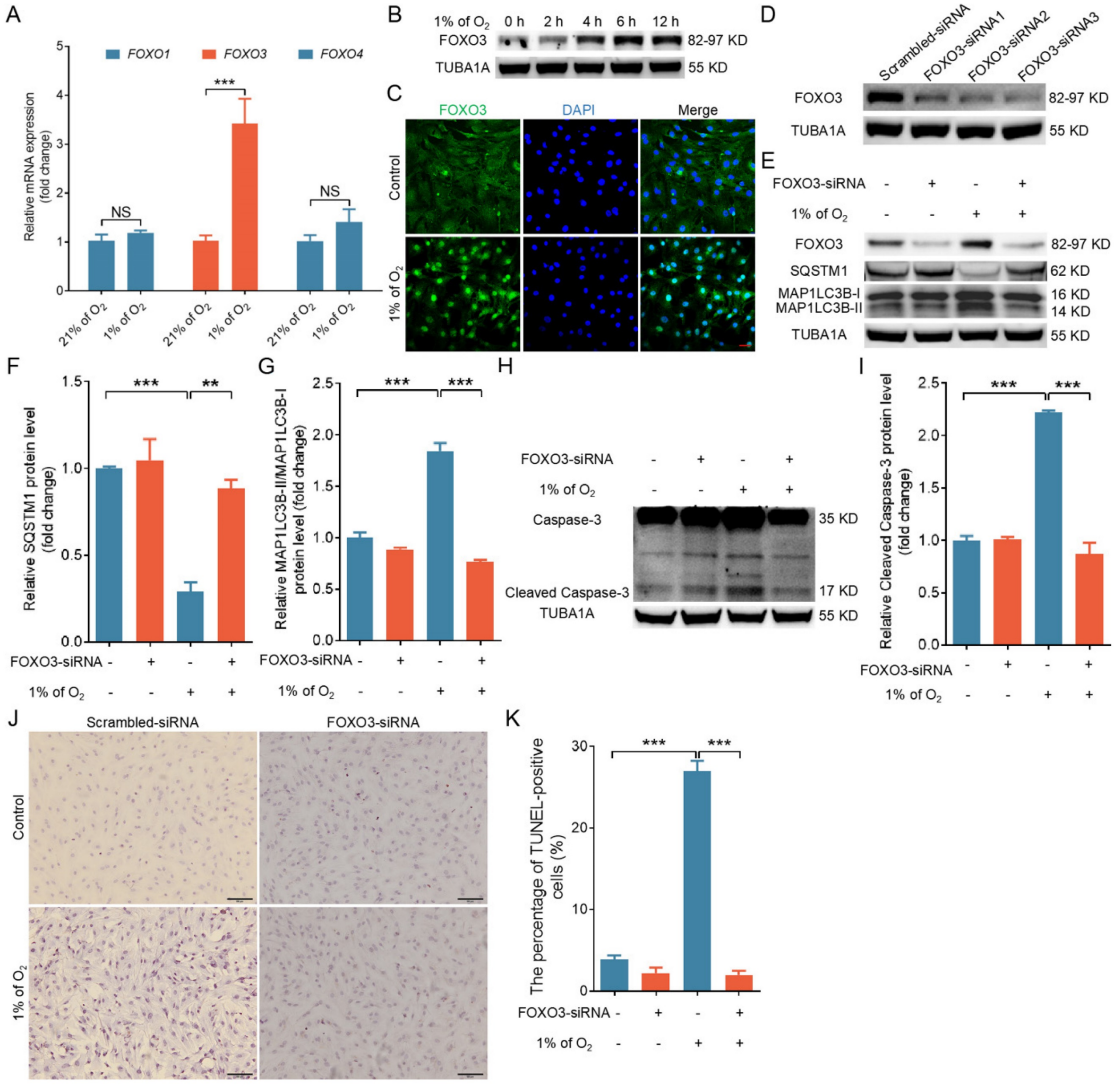
Hypoxia induces autophagic death by promoting the expression and nuclear import of FOXO3. **(A)** GCs underwent cultivation in either a normoxic environment with 21% oxygen or a hypoxic setting at 1% oxygen for a duration of 6 h. Subsequently, the expression levels of *FOXO1*, *FOXO3*, and *FOXO4* mRNAs were quantified using quantitative real-time polymerase chain reaction (qRT-PCR). **(B)** GCs were exposed to a low oxygen environment (1% O_2_) for varying durations, including 0, 2, 4, 6, and 12 h, and the protein expression of FOXO3 was analyzed through western blot. **(C)** GCs were incubated under either normal (21% O_2_) or low (1% O_2_) oxygen levels for 6 h before being harvested for analysis of FOXO3 subcellular localization using immunofluorescence staining. The scale bar indicates a length of 50 μm. **(D)** After being transfected with *FOXO3*-siRNA for 24 h, GCs were harvested to assess the silencing efficiency through western blot analysis. **(E-K)** After introducing either *FOXO3*-targeting siRNA or a non-targeting control into GCs for a 12-h period, the cells were further cultured for 12 h in environments with either normal (21% O_2_) or reduced (1% O_2_) oxygen levels. The abundance of proteins such as SQSTM1 and MAP1LC3B **(E)** along with cleaved caspase-3 **(H)** was analyzed via western blot. The protein levels were then measured using densitometry with ImageJ software **(F, G, and I)**. TUNEL assay was performed to detect the apoptosis rate **(J)**, and the percentage of TUNEL-positive cells was quantified **(K)**.

Not only FOXO1 but also other FOXO transcription factors are well established as crucial inducers of both autophagy and apoptosis in response to cellular stress 21. We identified a marked increase in *FOXO3* mRNA levels following hypoxic treatment by using qRT-PCR to analyze the expression of various FOXO family genes (Fig. 3A). Correspondingly, we observed that hypoxia significantly increased the protein expression of FOXO3 (Fig. 3B) and strongly enhanced its nuclear translocation (Fig. 3C and Fig. S4A, B). We knocked down FOXO3 expression with siRNA to investigate the potential role of FOXO3 in hypoxia-induced autophagic cell death (Fig. 3D). As depicted in Fig. 3E to K, *FOXO3* knockdown markedly inhibited hypoxia-induced autophagy (Fig. 3E-G and Fig. S4C, D) and apoptosis (Fig. 3H-K and Fig. S4C, D).

Insulin and IGF-I are known to suppress FOXO3 activity by preventing FOXO3 from entering into the cell nucleus 7. We applied IGF-I treatment to our GC model in order to confirm the role of FOXO3 in regulating GC autophagy/apoptosis under hypoxia. As shown in Fig. S5A, IGF-I administration successfully inhibited hypoxia-induced nuclear translocation of FOXO3. Consistent with the results obtained from RNA interference, IGF-I also abrogated the induction of autophagy (Fig. S5B-D) and apoptosis (Fig. S5E-G) during hypoxia. These results collectively indicate that the activation of FOXO3 is also required for hypoxia-mediated autophagy/apoptosis.

### FOXO1 triggers autophagic death by promoting FOXO3 expression

**Figure 4:**
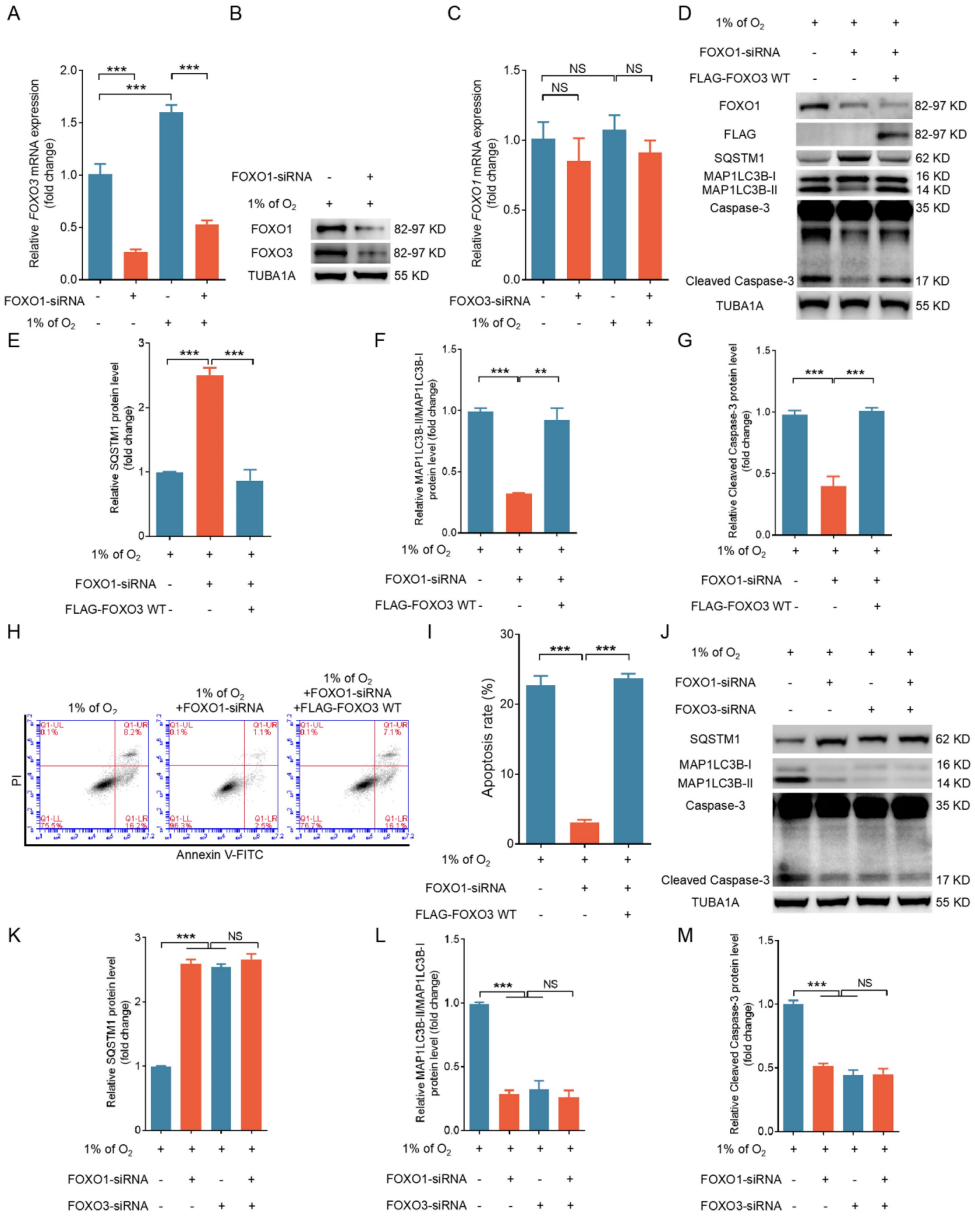
FOXO1 induces autophagic cell death in GCs by upregulating the expression of FOXO3. **(A-C)** Primary porcine GCs transfected with scramble control siRNA or siRNAs against* FOXO1* and *FOXO3* were subjected to a further 12-h incubation in environments with either 21% O_2_ (normoxia) or 1% O_2_ (hypoxia). The expression of *FOXO3* **(A)** and *FOXO1* **(B)** mRNA was analyzed using qRT-PCR and the protein expression of FOXO3 **(G)** using western blot analysis. **(D-I)** GCs treated with *FOXO1*-siRNA for 12 h were transfected with the FLAG-labeled FOXO3-WT plasmid and then incubated in a low-oxygen environment (1% O_2_) for periods of 12 and 24 h. The cells maintained in a low-oxygen environment for 12 h were retrieved to examine the protein expression of FOXO1, FLAG, SQSTM1, MAP1LC3B, or cleaved caspase-3 by western blot analysis **(D)**, followed by quantification using densitometry with ImageJ software **(E-G)**. After the cells were exposed to hypoxic conditions for 24 h, they were harvested for flow cytometry analysis to assess apoptosis **(H)**. The proportion of apoptotic cells was measured using flow cytometry **(I)**. **(J-M)** GCs were transfected with either *FOXO1*-siRNA, *FOXO3*-siRNA, or both for 12 h, followed by another 12 h culture under hypoxia. The cells were then harvested for protein level analysis of SQSTM1, MAP1LC3B, and cleaved caspase-3 through western blot **(J)**. The protein bands were quantified using densitometry with ImageJ software **(K-M)**.

Given the consistent and comparable regulatory patterns of FOXO1 and FOXO3 on apoptosis and autophagy in hypoxic GCs, it is conceivable that they might operate within a shared pathway. Indeed, studies have indicated that FOXO family members can reciprocally regulate transcription by binding to FRE within *FOXO* gene promoters 9-11. Because FOXO1 expression remained unchanged while FOXO3 expression increased during hypoxia exposure, it is more likely that FOXO1 acts as an upstream regulator of FOXO3 expression rather than the reverse. We used qRT-PCR to observe a substantial reduction in hypoxia-induced *FOXO3* transcription after knocking down *FOXO1* (Fig. 4A). FOXO1 depletion also blocked the protein expression of FOXO3 under hypoxia (Fig. 4B). In contrast, when we silenced *FOXO3* expression under hypoxia, we observed no significant alterations in FOXO1 transcription (Fig. 4C).

Next, we explored if FOXO1 acts through FOXO3 to mediate hypoxia-induced autophagic death. Enforced FOXO3 expression following FOXO1 depletion reactivated the autophagic/apoptotic signals triggered by hypoxia, as illustrated in Fig. 4D to I. Correspondingly, inhibition of FOXO1 and FOXO3 expression suppressed both autophagic and apoptotic signaling in hypoxic GCs; however, when FOXO1 was transcriptionally silenced, FOXO3 siRNA did not further reduce autophagy-related cell death in GCs (Fig. 4J-M). Therefore, these findings imply that FOXO1 may facilitate autophagic cell death by regulating FOXO3 expression.

### A mutant form of FOXO1 that cannot bind DNA enhances FOXO3 expression under hypoxia

**Figure 5:**
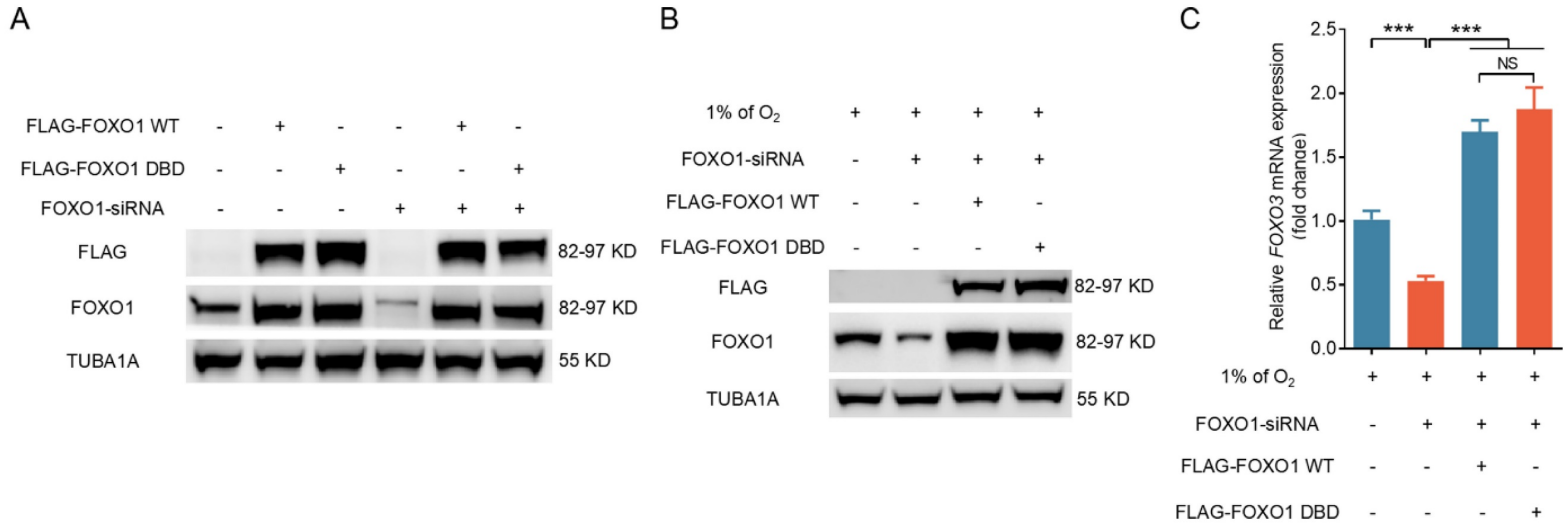
FOXO1 promotes FOXO3 expression without direct DNA binding under hypoxia. **(A-C)** GCs treated with *FOXO1*-siRNA for 12 h were transfected with FLAG-labeled FOXO1-expressing plasmids, including either wild-type FOXO1 (WT) or the FOXO1-DBD/FOXO1^N208A, H212R^ mutant. The cells were further cultured for 12 h in environments with either normal (21% O_2_) **(A)** or reduced (1% O_2_) **(B)** oxygen levels. Cell extracts were prepared for analysis of FLAG and FOXO1 protein levels through immunoblotting. The mRNA level of *FOXO3* was measured by qRT-PCR **(C)**.

To investigate further how FOXO1 regulates FOXO3 expression, we transfected GCs subjected to hypoxia exposure with plasmids expressing either wild-type FOXO1 (FOXO1-WT) or a FOXO1 mutant with two point mutations in the DNA-binding domain (FOXO1-DBD/FOXO1^N208A,H212R^) after silencing endogenous FOXO1. Western blot analysis results revealed a significant upregulation of FOXO1 in GCs following forced FOXO1 expression (Fig. 5A). RNA interference did not significantly affect the expression levels of FLAG-tagged FOXO1 in either the FOXO1-WT or the FOXO1-DBD groups, thus allowing us to focus on the specific effects of exogenously introduced FOXO1 (Fig. 5A). Western blot and qRT-PCR assays revealed that the overexpression of both FOXO1-WT and FOXO1-DBD significantly upregulated FOXO3 expression in porcine GCs, 293T cells, and NIH/3T3 cells pretreated with *FOXO1* siRNA (Fig. 5B, C and Fig. S6A, B). These results suggest that FOXO1 regulates FOXO3 expression without direct DNA binding in cells exposed to hypoxia.

### Hypoxia-induced nuclear import of FOXO1 transports STAT3 into the nucleus

**Figure 6:**
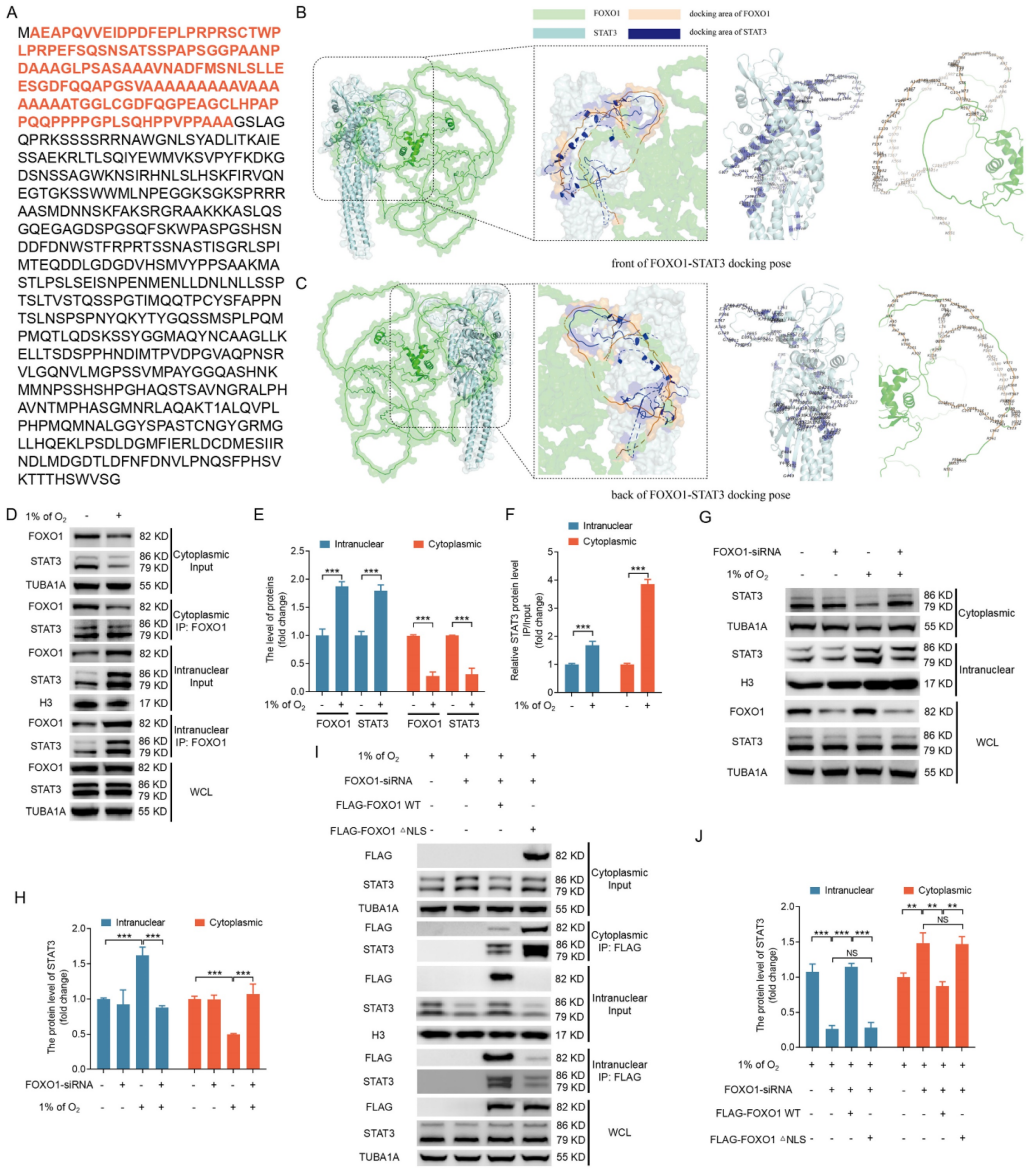
Hypoxia-induced nuclear translocation of FOXO1 facilitates the nuclear entry of STAT3. **(A-C)** The amino acid sequence of the STAT3 binding domain in the FOXO1 protein is highlighted in **(A)**. The front **(B)** and back **(C)** molecular docking diagrams of the three-dimensional structure of FOXO1 and STAT3 proteins are presented, with the amino acid sites on the interacting region of FOXO1 and STAT3 highlighted. **(D-F)** GCs were cultured for 12 h under either standard (21% O_2_) or reduced (1% O_2_) oxygen levels and subsequently subjected to nucleoplasmic extraction. The cytoplasmic and nuclear fractions of GCs were harvested to detect protein expression of FOXO1 and STAT3 by western blot and for immunoprecipitation (IP) analysis of the interactions between FOXO1 and STAT3 **(D)**. The intensity of the protein bands was measured through densitometric analysis using ImageJ software **(E and F)**. **(G and H)** GCs transfected with *FOXO1*-siRNA for 12 h were subsequently maintained in either normal (21% O_2_) or low (1% O_2_) oxygen levels for another 12 h. Cells were harvested to assess the protein levels of STAT3 in the nucleus and cytoplasm via western blot **(G)**, and the data were quantified using densitometry with ImageJ 1.42q software **(H)**. **(I and J)** GCs were treated with *FOXO1*-siRNA for 12 h before being transfected with plasmids encoding FLAG-labeled FOXO1 either in its wild-type configuration (WT) or a version with a mutated nuclear localization signal (△NLS). The transfected GCs were then maintained under low oxygen conditions (1% O2) for another 12 h. The cytoplasmic and nuclear fractions of GCs were collected for detecting protein levels of STAT3 by western blot and for IP analysis of the interactions between FOXO1 and STAT3 **(J)**. The protein levels were then measured using densitometry with ImageJ software **(J)**.

FOXO1-mediated FOXO3 expression does not require direct DNA binding, thus suggesting that FOXO1 forms complexes with other transcription factors to regulate FOXO3 expression. The transcription factor STAT3 has emerged as a potential interaction partner with FOXO1 via a query of the STRING database for FOXO1 binding proteins. Indeed, STAT3 has been reported to interact with the N-terminal 149 amino acids of murine FOXO1 22. We identified this region (highlighted in red) in porcine FOXO1 (Fig. 6A), with >84% sequence similarity among porcine, murine, and human cells (Fig. S7), via a pairwise alignment of the protein sequence of FOXO1.

To delve deeper into the intricate details of the interaction between STAT3 and FOXO1, we employed a combination of data from the Uniprot database (https://www.uniprot.org/) and AlphaFold2 software to predict the three-dimensional structures of porcine STAT3 and porcine FOXO1 proteins. We then executed molecular docking of the STAT3 and FOXO1 proteins using ClusPro software (version 2.0). We selected the binding pose with the lowest docking energy, indicative of the highest binding affinity between the two proteins, as the structural representation of their complex. We visualized the resulting docking output using Pymol software, with a specific focus on annotating key amino acid residues located at the interaction interface. The molecular docking results are depicted in Fig. 6B and C, which illustrate the three-dimensional structures of the STAT3-FOXO1 protein interaction from frontal and dorsal perspectives and the amino acid sequences involved in their mutual interaction. These results raise the possibility that FOXO1 may facilitate the nuclear entry of STAT3 to initiate FOXO3 transcription.

We examined the subcellular localization and interaction between FOXO1 and STAT3 under hypoxic conditions to explore this possibility. IP analysis of cytoplasmic and nuclear fractions confirmed an interaction between FOXO1 and STAT3, with hypoxia-induced FOXO1 nuclear localization coinciding with the nuclear translocation of STAT3 (Fig. 6D-F). We obtained consistent results when FOXO1 was knocked down, inhibiting the hypoxia-induced nuclear translocation of STAT3 (Fig. 6G, H), thus supporting the hypothesis that hypoxia acts through FOXO1 to facilitate STAT3 nuclear translocation.

STAT3 is known to be activated by phosphorylation at Tyr705, leading to its dimerization, nuclear translocation, and DNA binding 23. Here, we observed Tyr705 phosphorylation of STAT3 under normoxic and hypoxic conditions (Fig. S8); however, phosphorylated STAT3 failed to interact with FOXO1 under normoxia. In contrast, phosphorylated STAT3 forms an association with FOXO1 upon hypoxic exposure. This implies that the phosphorylation status of STAT3 is not an essential element in the interaction between STAT3 and FOXO1. In other words, FOXO1-mediated STAT3 nuclear translocation may not depend on STAT3 phosphorylation at Tyr705.

To further investigate the influence of FOXO1 nuclear transport on STAT3 subcellular localization, we transfected GCs, 293T cells, and NIH/3T3 cells grown in hypoxic conditions with FLAG-tagged vectors encoding FOXO1, including the FOXO1-WT and a mutant lacking the nuclear localization signal (FOXO1-△NLS) upon silencing endogenous FOXO1 expression. Immunoprecipitation analysis of cytoplasmic and nuclear fractions showed that FOXO1-WT interacted with STAT3 and enhanced the nuclear localization of STAT3. In contrast, the depletion of the STAT3-binding domain in FOXO1 disrupts the FOXO1-STAT3 interaction and inhibits the nuclear translocation of STAT3 (Fig. S9A, B). In addition, FOXO1-△NLS interacted with STAT3, but it failed to promote STAT3 nuclear translocation (Fig. 6I, J and Fig. S10A, B), emphasizing the essential role of FOXO1 nuclear entry under hypoxia as a prerequisite for targeting STAT3 into the nucleus.

### FOXO1-mediated nuclear translocation of STAT3 induces the transcriptional activation of FOXO3

**Figure 7:**
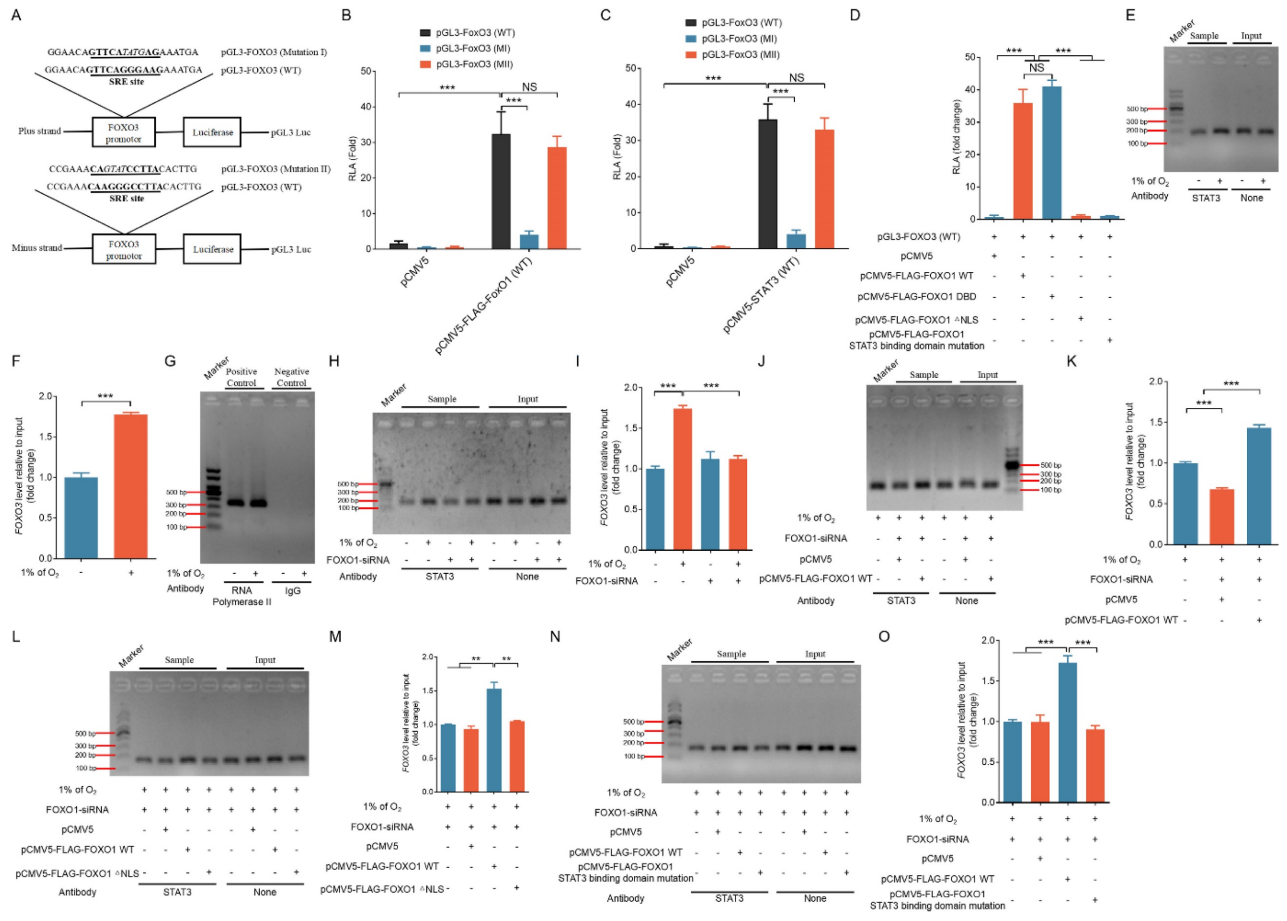
FOXO1-mediated nuclear transport of STAT3 activates the transcriptional expression of FOXO3. **(A)** Using murine genomic DNA as a template, a 2000 bp portion of the *FOXO3* promoter containing the SRE (CAAGGGCCTTA) sequence was amplified by PCR and inserted into the pGL3-Basic vector to generate the plasmid construct pGL3-FOXO3 (WT). Mutations were introduced into the SRE region to create the pGL3-FOXO3 constructs with Mutations I and II. **(B-D)** *FOXO3* reporter assays in GCs were performed after co-transfection with FOXO1 variants (FOXO1-WT, FOXO1-DBD, FOXO1-△NLS, or a STAT3 binding domain mutant) or STAT3-WT along with FOXO3 promoter constructs for 24 h. Reporter activity was normalized against pRL-TK. RLA refers to relative luciferase activity. **(E-G)** STAT3 binding to the *FOXO3* promoter in GCs was assessed using chromatin immunoprecipitation (ChIP) assays after exposure to either normoxic (21% O_2_) or hypoxic (1% O_2_) conditions for 12 h. DNA extracted from the immunoprecipitated complexes was employed as the template for qRT-PCR analysis. The resulting qRT-PCR products were separated on a 2% agarose gel **(E)** and quantified through densitometry using ImageJ software **(F)**. The GAPDH promoter immunoprecipitated with an RNA polymerase II antibody served as a positive control for the ChIP assay, and IgG was utilized as the negative control **(G)**. **(H and I)** GCs transfected with FOXO1-siRNA were incubated under either normoxic (21% O_2_) or hypoxic (1% O_2_) conditions for 12 h. ChIP assays were performed to assess STAT3 binding to the *FOXO3* promoter. The qRT-PCR products were resolved on a 2% agarose gel **(H)**, and densitometric analysis was conducted using ImageJ software **(I)**. After 12-h treatment with *FOXO1*-siRNA, FLAG-tagged vectors expressing FOXO1 variants—FOXO1-WT **(J and K)**, FOXO1-△NLS **(L and M)**, and the STAT3-binding domain mutant **(N and O)**—were introduced into GCs. The cells were subsequently subjected to hypoxic conditions (1% O_2_) for another 12 h before DNA was extracted from the precipitated complexes and used as a template for qRT-PCR. The PCR products were separated on a 2% agarose gel and quantified using densitometric analysis with ImageJ software.

After that, we proceeded to assess whether FOXO1-mediated nuclear translocation of STAT3 could activate FOXO3 expression at the promoter level. We queried the JASPAR database and we identified two SRE motifs (GTTCAGGGAAG and CAAGGGCCTTA) within the *FOXO3* promoter region. Consequently, we inserted a portion of the 5' untranslated region (UTR) of *FOXO3* containing either the predicted SRE site or its mutated versions ahead of the luciferase reporter gene, generating the constructs pGL-FOXO3 (WT), pGL-FOXO3 (MI), and pGL-FOXO3 (MII) (Fig. 7A).

We co-transfected GCs with either pCMV5-FLAG-FOXO1 (FOXO1-WT), pCMV5-STAT3 (STAT3-WT), or the control pCMV5 together with pRLTK. Figure 7B and C demonstrate that the activation of *FOXO3* promoter was significantly elevated following the introduction of STAT3-WT and FOXO1-WT vectors. Mutating the type I SRE site (MI) significantly diminished the luciferase reporter's sensitivity to the overexpression of FOXO1 and STAT3. Later investigations involving co-transfection with various FOXO1 expression vectors in GCs, 293T cells, and NIH/3T3 cells demonstrated that co-transfection with both FOXO1-WT and FOXO1-DBD significantly promoted FOXO3 promoter activity, whereas co-transfection with FOXO1-△NLS and the FOXO1 mutant lacking STAT3 binding domain was ineffective (Fig. 7D and Fig. S11A and B). These findings suggest the existence of collaboration between STAT3 and FOXO1 in nuclear translocation and binding to the FOXO3 promoter for the activation of FOXO3 transcriptional expression.

We then conducted ChIP analysis by fragmenting GC chromatin using micrococcal nuclease for employment in ChIP assays in order to determine whether STAT3 interacts with the FOXO3 promoter. As depicted in Fig. 7E and F, the STAT3 antibody enriched the FOXO3 promoter area surrounding the SRE site, and RNA Polymerase II and IgG antibodies were used as positive and negative controls, respectively (Fig. 7G). Of note, the signals showed a pronounced increase in GCs under hypoxia treatment (Fig. 7E and F), but they were substantially attenuated in hypoxic GCs after knockdown of FOXO1 (Fig. 7H and I).

Additional co-transfection experiments with various FOXO1 expression vectors revealed that both FOXO1-WT and FOXO1-DBD facilitated the binding of STAT3 to the *FOXO3* promoter (Fig. 7J, K and Fig. S12A, B), whereas FOXO1-△NLS and the FOXO1 mutant lacking STAT3 binding domain were ineffective (Fig. 7L, M; Fig. S9; and Fig. 7N, O). Consistent with these results, in GCs, 293T cells, and NIH/3T3 cells, both FOXO1-WT and FOXO1-DBD markedly upregulated the expression of cleaved caspase-3 and MAP1LC3B-II, whereas FOXO1-△NLS and the FOXO1 mutant lacking STAT3 binding domain showed no discernible effect (Fig. S13A-C). These findings suggest that FOXO1 is recruited directly to the *FOXO3* promoter, where it collaborates with STAT3, thereby activating *FOXO3* transcription and subsequently triggering apoptotic death under hypoxia.

We randomly selected three FOXO1 and three STAT3 target genes from normoxic and hypoxic conditions from the differentially expressed genes that were identified in RNA-seq results (unpublished data). ChIP-qPCR experiments using STAT3 or FOXO1 antibodies revealed that under hypoxia, anti-FOXO1 not only precipitated the promoters of FOXO1 target genes but also captured the promoter sequences of STAT3 target genes (Fig. S14A). Similarly, anti-STAT3 demonstrated the ability to precipitate the promoter sequences of STAT3 target genes as well as those of FOXO1 target genes (Fig. S14B). These results suggest that the formation of the FOXO1-STAT3 complex is essential for the transcriptional activation of both FOXO1 and STAT3 target genes. Further amplification of the *FOXO3* promoter region using primers targeting the FRE and SRE revealed that both anti-STAT3 and anti-FOXO1 could precipitate the SRE region (Fig. S14A, B and Fig. S15A-D) in GCs, 293T cells, and NIH/3T3 cells but that neither could precipitate the FRE region (Fig. S14C), indicating that the FRE site within the *FOXO3* promoter cannot be recognized by FOXO1.

### Formation of the FOXO1-AKT1 complex promotes the nuclear localization of AKT1 under hypoxia

Apart from investigating the mechanisms governing the upregulation of FOXO3 expression, another pivotal aspect of this study was unraveling the molecular processes controlling FOXO3 nuclear translocation under hypoxic conditions. Although it is widely acknowledged that AKT1 activation typically inhibits the nuclear translocation of FOXO3 7, our prior work uncovered a noteworthy increase in AKT1 activity under hypoxia, as evidenced by elevated phosphorylation levels at serine 473 18. This appears to contradict our observation of the promotion of FOXO3 nuclear translocation under hypoxia in the current study. Based on predictions from the STRING database, we hypothesized that AKT1 can interact with FOXO1 protein. This hypothesis led us to further speculate that under hypoxia, AKT1 enters the nucleus by forming a complex with FOXO1, thereby preventing AKT1 from catalyzing the nuclear exclusion of FOXO3.

**Figure 8:**
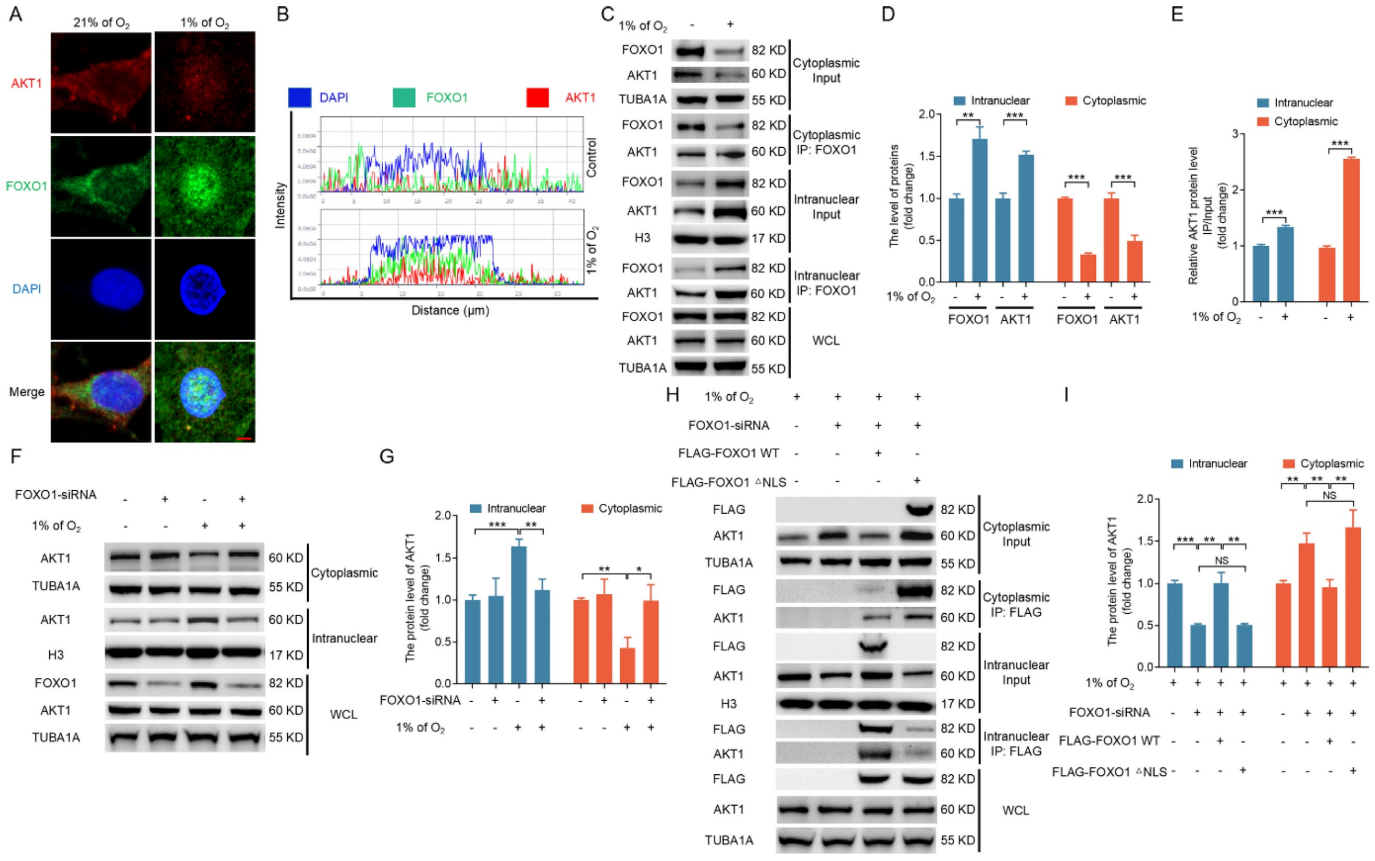
FOXO1 forms a complex with AKT1 and facilitates AKT1 nuclear translocation under hypoxia. **(A-E)** Cells were collected to assess the co-localization of FOXO1 and AKT1 by laser confocal-scanning microscopy **(A)**. Bar, 2 μm. The fluorescence intensity curve illustrates the spatial distributions of AKT1 (red), FOXO1 (green), and DAPI (blue) across cells **(B)**. The cytoplasmic and nuclear fractions of GCs were retrieved to examine protein levels of FOXO1 and AKT1 by western blot and for immunoprecipitation (IP) analysis of the interactions between FOXO1 and AKT1 **(C)**. The protein levels were then measured using densitometry with ImageJ software **(D and E)**. **(F and G)** After treatment with FOXO1-siRNA for 12 h, GCs were cultured under normoxia (21% O_2_) or hypoxia (1% of O_2_) for 12 h. Cells were collected for measuring STAT3 protein levels in the nucleus and cytoplasm via western blot **(F)**, followed by quantification using densitometry analysis with ImageJ software **(G)**. **(H and I)** After 12 h of *FOXO1*-siRNA treatment, GCs were transfected with FLAG-tagged FOXO1-expressing plasmids, including both FOXO1-WT and FOXO1-△NLS constructs. The transfected cells were then subjected to hypoxia (1% O_2_) for another 12 h. The cytoplasmic and nuclear fractions of GCs were collected for detecting protein levels of AKT1 by western blot and for IP analysis of the interactions between FOXO1 and AKT1 **(H)**. The protein levels were then measured using densitometry with ImageJ software **(I)**.

To explore this potential interaction, we investigated the subcellular localization and dynamic interplay between FOXO1 and AKT1 during hypoxic conditions. Immunofluorescence staining revealed an accumulation of both FOXO1 and AKT1 in the nucleus upon hypoxic stimulation (Fig. 8A, B). Further supporting this observation, IP analysis of cytoplasmic and nuclear fractions confirmed a direct interaction between FOXO1 and AKT1. Notably, the hypoxia-induced nuclear localization of FOXO1 coincided with the nuclear translocation of AKT1 (Fig. 8C-E). We obtained consistent results when FOXO1 was knocked down, which led to the inhibition of hypoxia-induced nuclear translocation of AKT1 (Fig. 8F, G). These findings substantiate the proposition that hypoxia operates through FOXO1 to facilitate the nuclear translocation of AKT1.

To further verify whether FOXO1 nuclear translocation affects the subcellular positioning of AKT1, we introduced vectors into cells grown in a hypoxic environment carrying FLAG-tagged FOXO1 constructs, including both the FOXO1-WT and the NLS-deleted FOXO1 variant, after knockdown of the endogenous FOXO1. IP analysis of cytoplasmic and nuclear fractions in GCs, 293T cells, and NIH/3T3 cells revealed that FOXO1-WT not only interacted with AKT1 but also promoted the nuclear localization of AKT1. Although FOXO1-△NLS also interacted with AKT1, it failed to facilitate the nuclear translocation of AKT1 (Fig. 8H, I and Fig. S16A, B), underscoring the pivotal role of FOXO1 nuclear entry for guiding AKT1 into the nucleus.

### FOXO1 mediates hypoxia-induced nuclear localization of FOXO3 by sequestering AKT1 in the nucleus

**Figure 9:**
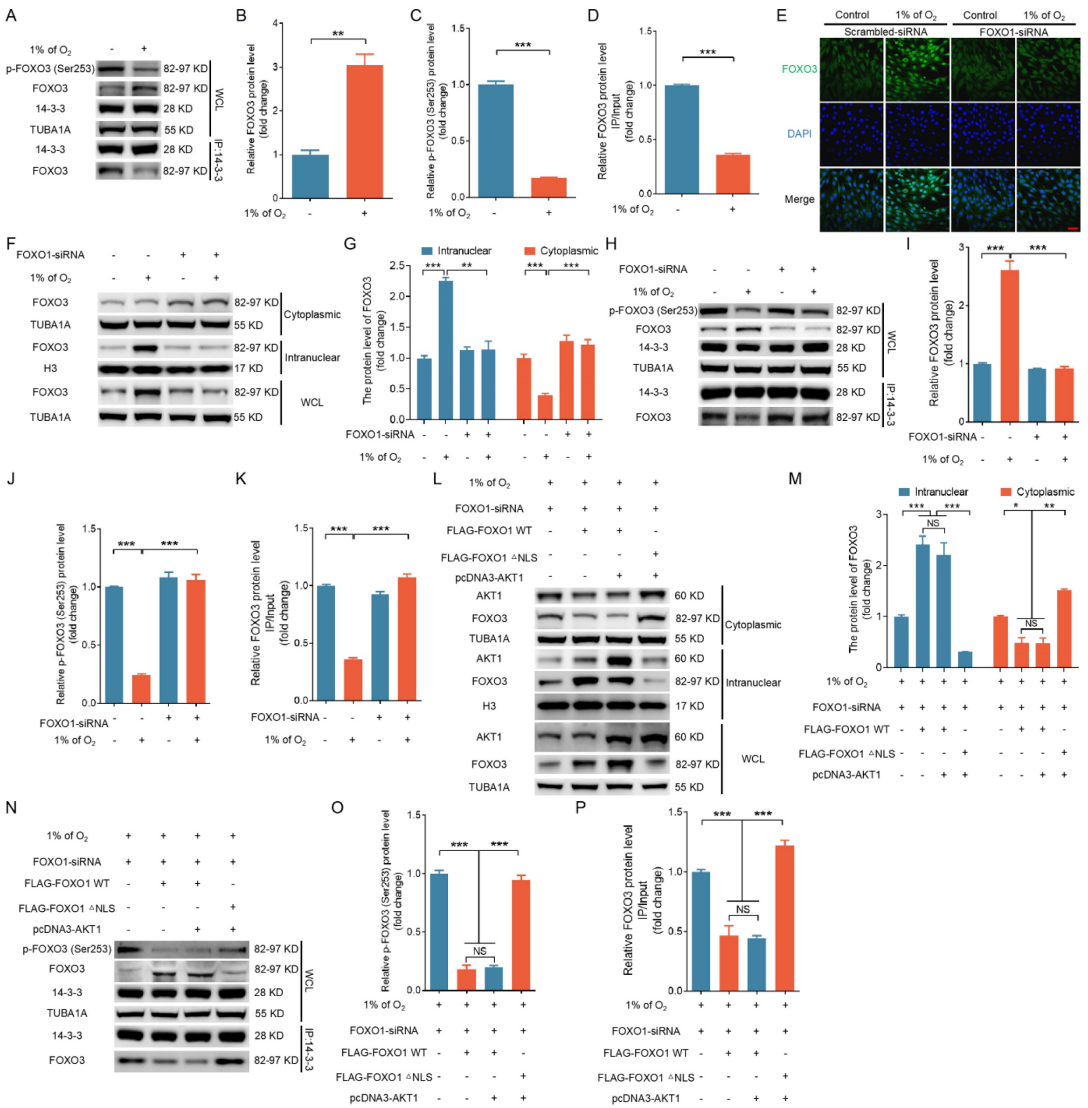
FOXO1-mediated AKT1 nuclear localization induces nuclear transportation of FOXO3 under hypoxia. **(A-D)** GCs were cultured for 12 h under either standard (21% O_2_) or reduced (1% O_2_) oxygen levels. Cell lysates were then collected to examine the expression of p-FOXO3 (Ser253) or the interaction between FOXO3 and 14-3-3 **(A)**, and the data were quantified using densitometry with ImageJ 1.42q software **(B-D)**. **(E-K)** After introducing either *FOXO1*-targeting siRNA or a non-targeting control into GCs for a 12-h period, the cells were cultured for a further 12 h in environments with either normal (21% O_2_) or reduced (1% O_2_) oxygen levels. The subcellular distribution of FOXO3 was then examined using laser confocal scanning microscopy **(E)**. Bar, 50 μm. The cytoplasmic and nuclear fractions of GCs were collected for detecting protein levels of FOXO3 by western blot **(F)**, followed by quantification using densitometry analysis with ImageJ 1.42q software **(G)**. The expression of p-FOXO3 (Ser253) or the interaction between FOXO3 and 14-3-3 was determined by western blot or IP, respectively **(H)**. The protein levels were then measured using densitometry with ImageJ software **(I-K)**. **(L-P)** GCs treated with FOXO1-siRNA for 12 h were transfected with FLAG-labeled FOXO1-expressing plasmids, including either FOXO1-WT or FOXO1-△NLS mutant with or without AKT1 expression vector and then cultured under hypoxic (1% O_2_) conditions for 12 h. The protein levels of FOXO3 and AKT1 in the nucleus or cytoplasm were examined by western blot **(L)**, followed by quantification using densitometry analysis with ImageJ 1.42q software **(M)**. The expression of p-FOXO3 (Ser253) and the interaction of FOXO3 and 14-3-3 were determined by immunoblotting or IP, respectively **(N)**. The protein levels were then measured using densitometry with ImageJ software **(O and P)**.

Activated AKT1 interacts with FOXO3 to phosphorylate Ser253, which inhibits the association of FOXO3 with 14-3-3 protein and promotes the nuclear exclusion of FOXO3 7. We examined whether FOXO1-mediated nuclear distribution of AKT1 would affect the binding between 14-3-3 and FOXO3 under hypoxia. As shown in Fig. 9A to D, 14-3-3 was pulled down using an antibody against FOXO3. The affinity between 14-3-3 and FOXO3 was notably diminished following treatment with 1% oxygen, coinciding with a reduction in FOXO3 phosphorylation at Ser253. Immunofluorescence staining and nuclear-cytoplasmic fractionation experiments revealed that the knockdown of FOXO1 inhibited hypoxia-induced nuclear translocation of FOXO3 (Fig. 9E-G). Immunoprecipitation reactions using the 14-3-3 antibody indicated that FOXO1 knockdown counteracted the inhibitory effects of hypoxia on FOXO3 phosphorylation and reinstated the interaction between 14-3-3 and FOXO3 (Fig. 9H-K).

To further validate the functional significance of AKT1-FOXO1 nuclear translocation in regulating FOXO3 subcellular localization, we introduced GCs maintained in a low-oxygen environment with plasmids carrying either FOXO1-WT or a mutated version of FOXO1 without its nuclear localization signal (FOXO1-△NLS), along with an AKT1-expressing vector (pcDNA3-AKT1), following the knockdown of endogenous FOXO1. IP analysis of cytoplasmic and nuclear fractions in GCs, 293T cells, and NIH/3T3 cells revealed that even with AKT1 overexpression, FOXO1-WT continued to facilitate AKT1 nuclear translocation, which was associated with the nuclear localization of FOXO3 (Fig. 9L, M and Fig. S17A). In contrast, FOXO1-△NLS failed to induce AKT1 nuclear translocation and was accompanied by decreased FOXO3 nuclear localization (Fig. 9L, M and Fig. S17A).

Subsequent investigations revealed that even in the context of AKT1 overexpression, FOXO1-WT retained its capacity to enhance FOXO3 protein expression, mitigate Ser353 phosphorylation of FOXO3, and disrupt the interaction between 14-3-3 and FOXO3 proteins (Fig. 9N-P and Fig. S17B). In contrast, FOXO1-△NLS failed to counteract the effects of AKT1 overexpression on FOXO3 expression, phosphorylation, and its interaction with 14-3-3 (Fig. 9N-P and Fig. S17B). These data suggest that the nuclear sequestration of AKT1 by FOXO1 prevents FOXO3 from AKT1-mediated phosphorylation, thereby suppressing 14-3-3/FOXO3 interaction and facilitating FOXO3 nuclear translocation.

### FOXO3 triggers ATG3-dependent autophagy and apoptosis upon hypoxic exposure

**Figure 10:**
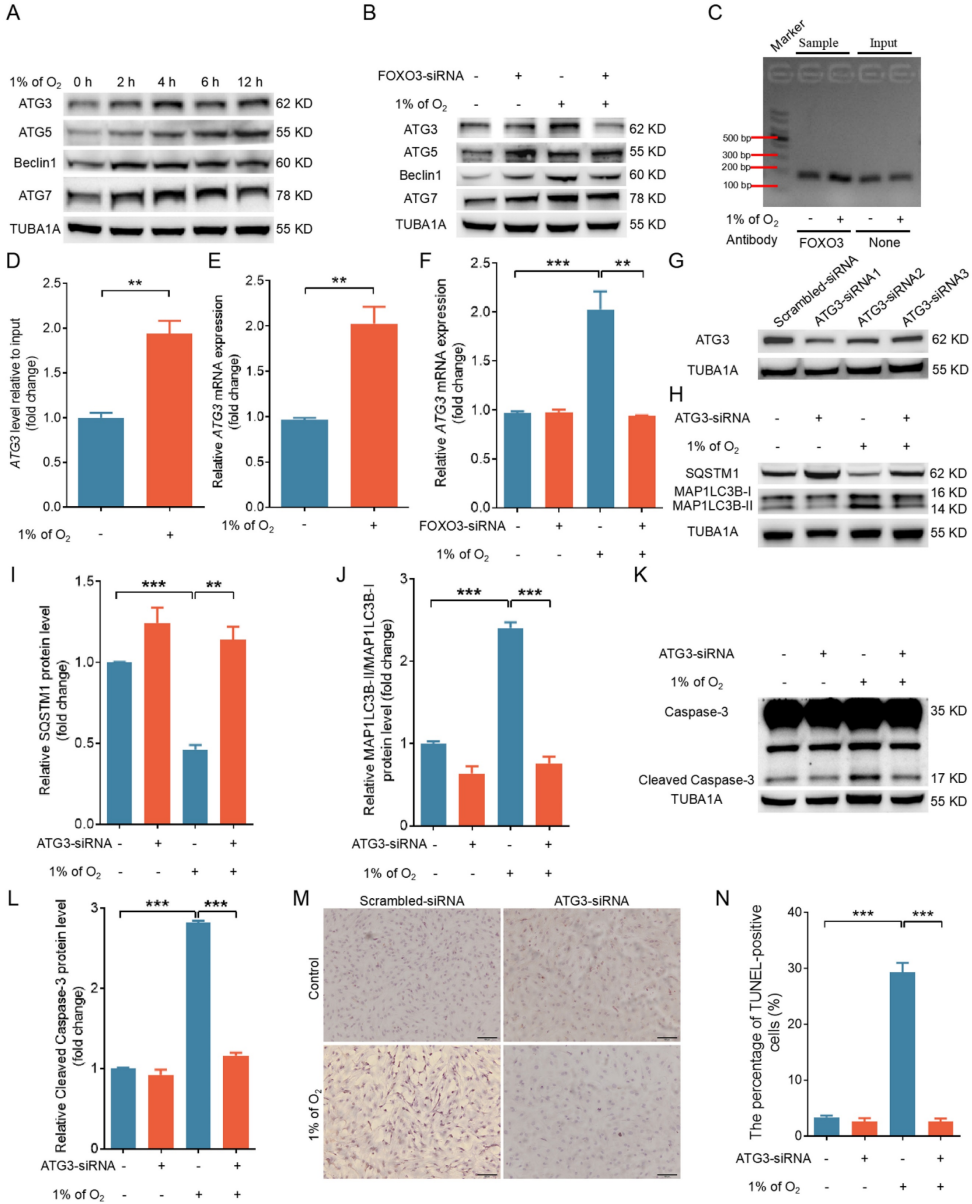
FOXO3 acts through ATG3 to promote autophagy and autophagy-dependent apoptosis upon hypoxia exposure. **(A)** GCs were grown with 1% O_2_ for 0, 2, 4, 6, or 12 h, and western blot was performed to determine the levels of autophagy-related proteins, including ATG3, ATG5, Beclin1, and ATG7. **(B)** GCs treated with FOXO3-siRNA were cultured under normoxia (21% O_2_) or hypoxia (1% O_2_) for 12 h, followed by immunoblotting analysis of the expression levels of ATG3, ATG5, Beclin1, and ATG7. **(C and D)** ChIP assay utilizing the FOXO3 antibody was performed to assess the interaction of FOXO3 with the ATG3 promoter in GCs subjected to either normoxic (21% O_2_) or hypoxic (1% O_2_) conditions for 12 h. DNA recovered from the complexes provided the template for subsequent qRT-PCR assays. The amplified qRT-PCR fragments were evaluated using a 2% agarose gel **(C)** and measured for density with ImageJ software **(D)**. **(E)** For 12 h, GCs were exposed to conditions of standard (21% O_2_) or reduced (1% O_2_) oxygen, after which the expression levels of *ATG3* mRNA were evaluated using qRT-PCR. **(F)** GCs transfected with *FOXO3*-siRNA were incubated under either normoxic (21% O_2_) or hypoxic (1% O_2_) conditions for 12 h, after which *ATG3* mRNA expression was measured via qRT-PCR analysis. **(G)** After transfection with *ATG3*-siRNA for 24 h, GCs were collected to determine ATG3 levels by western blot. **(H-N)** GCs were transfected with either *ATG3*-siRNA or a non-targeting siRNA control for 12 h, followed by culturing for another 12 or 24 h under conditions of either normoxia (21% O_2_) or hypoxia (1% O_2_). Following another 12-h incubation period, the cells were collected to evaluate the protein expression of SQSTM1, MAP1LC3B **(H)**, and the activated form of caspase-3 **(K)** by western blot. The data were quantified using densitometry with ImageJ 1.42q software **(I, J, and L)**, the apoptosis rate was evaluated using TUNEL staining **(M)**, and the percentage of TUNEL-positive cells was quantified **(N)**.

As a transcription factor, FOXO3 has been shown to promote autophagy by upregulating the expression of autophagy-related genes 24. To gain further insight into how FOXO3 promotes autophagy under hypoxia, we examined the expression of several autophagy-related proteins. Our findings revealed a substantial increase in the protein levels of ATG3, ATG5, Beclin1, and ATG7 under hypoxia (Fig. 10A). Notably, silencing FOXO3 selectively hindered the hypoxia-induced upregulation of ATG3 while leaving the expression of the aforementioned proteins unaffected (Fig. 10B and Fig. S18A, B). Using ChIP assay and qRT-PCR analysis, we observed that hypoxia exposure could facilitate the binding of FOXO3 to the *ATG3* promoter, which was accompanied by a significant elevation in *ATG3* transcription levels (Fig. 10C-E). In contrast, knockdown of FOXO3 markedly suppressed hypoxia-induced mRNA expression of *ATG3* (Fig. 10F). These results align with the results obtained when IGF-I was introduced under hypoxic conditions (Fig. S19), suggesting that FOXO3 activates autophagy by specifically promoting ATG3 expression under hypoxia.

ATG3 is an E2-like conjugating enzyme that facilitates the lipidation of MAP1LC3B-I to generate MAP1LC3B-II. Assessing the essential role of ATG3 in hypoxia-induced autophagy, we employed RNA interference to knock down *ATG3* (Fig. 10G and Fig. S18A, B) and observed a marked reduction in the protein level of MAP1LC3B-II (Fig. 10H-J and Fig. S18A, B). This reduction coincided with a significant decline in cleaved caspase-3 protein expression and apoptosis rates under hypoxia (Fig. 10K-N and Fig. S18A, B). These findings collectively suggest that hypoxia, acting through the FOXO3-ATG3 signaling axis, instigates autophagic cell death.

### *In vivo* evidence supports the relevance of the FOXO1-FOXO3 axis in mediating autophagic cell death in porcine ovarian GCs

**Figure 11:**
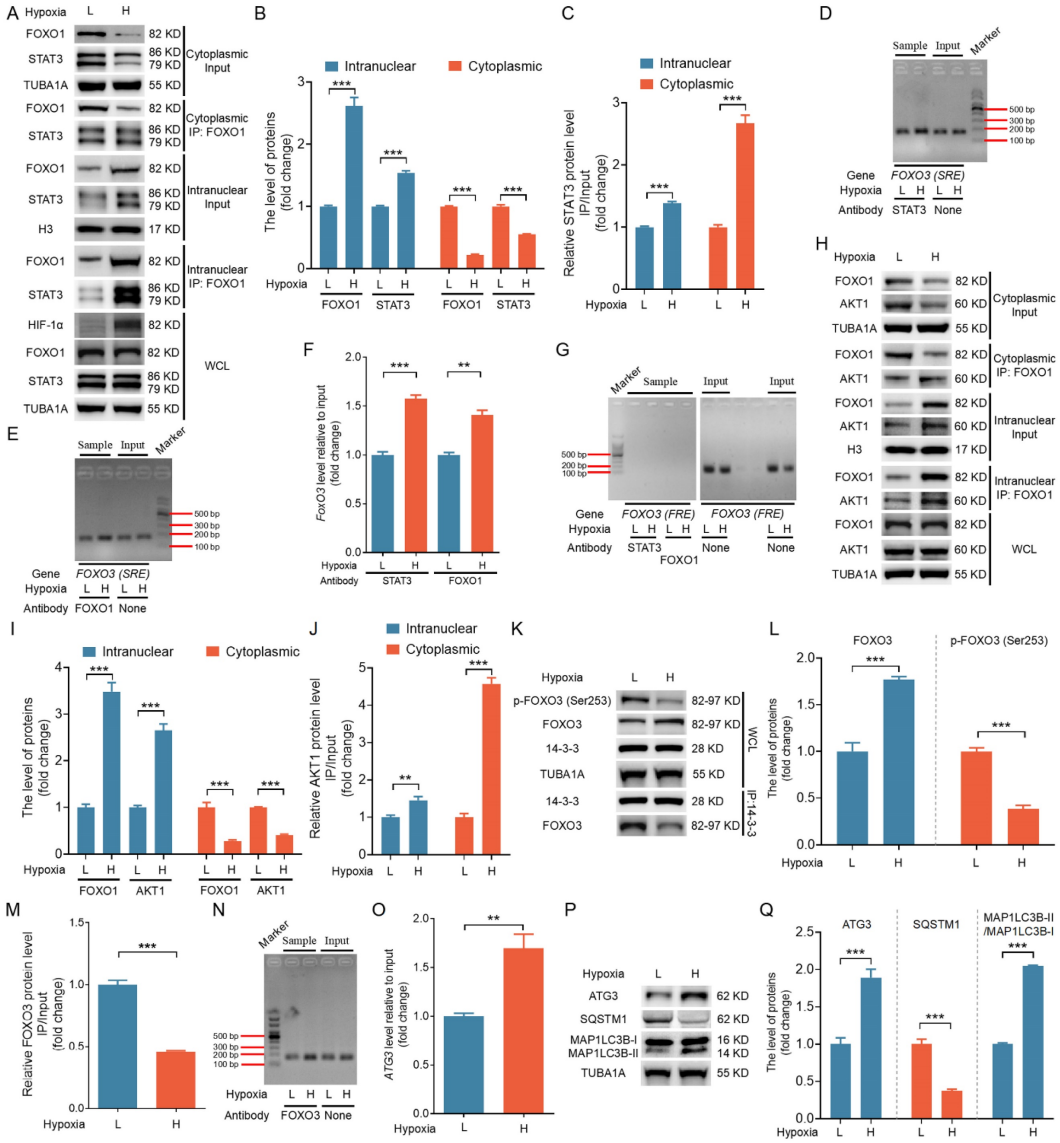
*In vivo* verification of the mechanistic model for hypoxia-induced autophagic GC death through the FOXO1-FOXO3 axis. GCs were isolated from 26 porcine ovarian follicles (~4 mm in diameter) and categorized into low **(L)** and high **(H)** hypoxia groups based on HIF-1α protein levels. **(A-C)** After nucleoplasmic separation, the cytoplasmic and nuclear fractions were subjected to western blot analysis to assess the expression of FOXO1 and STAT3 **(A and B)**. IP assays were conducted to evaluate the interaction between FOXO1 and STAT3 **(A and C)**. **(B and C)** Densitometric quantification of protein bands in **(A)** was performed using ImageJ 1.42q software. **(D-G)** ChIP assays were conducted using antibodies against STAT3 or FOXO1. qRT-PCR was performed to amplify the FOXO3 promoter regions containing either the SRE **(D and E)** or FRE **(G)** motifs. PCR amplicons were separated on a 2% agarose gel, and their intensity was measured via densitometry using ImageJ software **(F)**. **(H-J)** Western blot and IP analyses were performed on cytoplasmic and nuclear fractions to assess FOXO1 and AKT1 protein levels and their interactions **(H)**. The protein levels were then measured using densitometry with ImageJ software **(I and J)**. **(K-M)** Whole-cell lysates were analyzed by western blot to measure p-FOXO3 (Ser253) and FOXO3 levels, as well as the interaction between FOXO3 and 14-3-3 **(K)**. Densitometric analysis of the data was conducted using ImageJ 1.42q software **(L and M)**. **(N and O)** ChIP assay was conducted using the FOXO3 antibody to investigate its binding to the ATG3 promoter. DNA isolated from the immunoprecipitates was utilized as the input for PCR, and the amplification products were detected on a 2% agarose gel **(N)** and measured using densitometric analysis **(O)**. **(P and Q)** Western blot analysis was performed to measure the levels of autophagy-related proteins, including ATG3, SQSTM1, and MAP1LC3B. The protein levels were then measured using densitometry with ImageJ software.

To assess whether the *in vitro* mechanisms also occur *in vivo*, we isolated 26 follicles, each approximately 4 mm in size, from porcine ovaries. After extracting and categorizing GCs into low (L) and high (H) hypoxia groups based on HIF-1α protein levels, we obtained nuclear and cytoplasmic fractions through nucleoplasmic separation followed by immunoblotting and IP analyses. As shown in Fig. 11A to C, FOXO1 and STAT3 were more abundant in the nuclear fractions of the H group, with a notable increase in STAT3 binding to FOXO1 compared to the L group. ChIP assay revealed that both STAT3 and FOXO1 exhibited enhanced binding to the SRE motifs in the H group relative to the L group (Fig. 11D-F). However, neither protein showed binding to the FRE motif (Fig. 11G).

Western blot analysis showed significantly elevated FOXO3 protein levels in the H group compared with the L group (Fig. 11K, L). GCs from the H group exhibited elevated nuclear levels of AKT1, along with enhanced binding of FOXO1 to AKT1, in both the nuclear and cytoplasmic fractions compared with the L group (Fig. 11H-J). IP assays revealed a significant reduction in the binding of FOXO3 to 14-3-3 and lower levels of p-FOXO3 (Ser253) in the H group compared with the L group (Fig. 11K-M). ChIP assay performed to confirm the role of FOXO3 as a transcription factor promoting ATG3 expression *in vivo* showed that FOXO3 bound more efficiently to the ATG3 promoter sequences in the H group compared with the L group (Fig. 11N, O). Further, both ATG3 protein levels and overall autophagy levels were significantly elevated in the H group relative to the L group (Fig. 11P-Q). Together, these findings provide *in vivo* evidence supporting the relevance of the FOXO1-FOXO3 axis in mediating hypoxia-induced autophagic death.

## Discussion

Prior research has demonstrated the ability of FOXO1 to regulate its own transcription 9 and has shown FOXO3 can activate both the FOXO1 and FOXO3 promoters 10, 11. However, there has been no documented evidence that FOXO1 can influence FOXO3 transcription, largely due to the prevailing notion that the FOXO3 promoter region lacks the FOXO1-binding motif known as the FOXO-recognized element (FRE) 25. Contrary to this hypothesis, our data reveal the presence of an FRE sequence within the porcine *FOXO3* promoter region from -549 bp to -568 bp. Further investigation involving the amplification of the *FOXO3* promoter using primers targeting the FRE region showed that anti-FOXO1 failed to precipitate the FRE sequence, thus suggesting that FOXO1 cannot recognize the FRE site within the FOXO3 promoter.

Our findings indicate that FOXO1 can upregulate FOXO3 transcription without direct DNA binding under hypoxic conditions. Indeed, FOXO1 forms a complex with the transcription factor STAT3, translocates into the nucleus, and binds to the SRE within the FOXO3 promoter. ChIP-qPCR experiments using STAT3 antibodies further revealed that under hypoxia, anti-STAT3 not only precipitated the promoters of STAT3 target genes, but it also captured the promoter sequences of FOXO1 target genes. These results suggest that the binding of STAT3 to FOXO1 may represent a previously unrecognized mechanism for activating FOXO1 transcriptional activity in low-oxygen environments.

The canonical transcriptional activity of STAT3 primarily relies on phosphorylation at Tyr705, which determines the formation of functional dimers, nuclear localization, and DNA binding capacity 23. However, STAT3 has been hypothesized to continuously translocate to the nucleus by associating with particular import carriers, specifically importins, and not to rely on tyrosine phosphorylation 26. Unphosphorylated STAT3 has been shown to shuttle dynamically between the nucleus and cytoplasm 27 and interact with other factors, as well as directly or indirectly bind to specific DNA target sites 28-30.

Here, in this study, we demonstrated that FOXO1-mediated STAT3 nuclear transport is independent of STAT3 phosphorylation at Tyr705. The depletion of FOXO1 or deletion of its nuclear localization signal (NLS) or STAT3 binding domain significantly impaired the hypoxia-induced upregulation of STAT3 nuclear import. Further, we revealed that the formation of the FOXO1-STAT3 complex is essential for the transcriptional activation of STAT3 target genes. These findings collectively suggest that FOXO1 is a crucial determinant in governing STAT3 transcriptional activity in the context of hypoxia.

AKT1 has been documented to translocate into the nucleus in response to various stimuli, including insulin-like growth factor-I (IGF-I), the F(ab')2 fragment of anti-mouse IgG targeting the B-cell receptor, hypoglycemia, insulin, and nerve growth factor (NGF) 31. However, AKT1 lacks a canonical NLS or other recognized shuttling sequence, and the precise mechanism underlying its nuclear translocation remains elusive. Indeed, some substrates of AKT1 are localized in the nucleus, such as the FOXO family of transcription factors 32 and the transcriptional coactivator p300 33. This prompted us to question whether certain AKT1 substrates influence the shuttling of AKT1 between the cytosol and nucleoplasm. It is well established that AKT1-mediated phosphorylation promotes nuclear exclusion of the FOXO proteins 7, but there is a gap in the literature regarding whether FOXOs reciprocally affect the nuclear localization of AKT1. Here, we provide the first evidence demonstrating that FOXO1 associates with AKT1 and facilitates its translocation into the nucleus under hypoxic conditions. This discovery not only offers a potential explanation for understanding the mechanism of AKT1 nuclear translocation but also introduces a model of the bidirectional influence between AKT1 and FOXO1, challenging conventional models of unidirectional signaling.

FOXO1 and FOXO3, both members of the FOXO transcription factor family, share common upstream regulatory pathways in terms of their activity regulation 7. Our prior work revealed that despite increased AKT1 activity under hypoxia, FOXO1 remained confined within the nuclei of cells under hypoxic conditions 18. Typically, the phosphorylation of FOXO1 by AKT1 forms docking sites for 14-3-3 proteins, aiding in the translocation of FOXO1 out of the nucleus 7. This phenomenon indicates that hypoxic conditions may hinder the interaction of FOXO1 with 14-3-3. In fact, we identified JNK1/2 to be a principal initiator of FOXO1 activation under hypoxia. Notably, JNK1/2 facilitated the entry of FOXO1 into the nucleus by phosphorylating 14-3-3 at Ser184/186, thereby preventing the binding of 14-3-3 to FOXO1 18. This result offers a possible reason for the inability of AKT1 to affect the translocation of FOXO1 between the cytoplasm and nucleus upon hypoxia exposure. Intriguingly, we observed that under hypoxic conditions, FOXO1 sequesters AKT1 into the nucleus, reducing FOXO3 phosphorylation by AKT1 at Ser253. This reduction in phosphorylation impairs the binding of FOXO3 with 14-3-3, thereby causing the constitutive nuclear distribution of FOXO3. Therefore, in contrast to FOXO1, the nuclear-cytoplasmic shuttling of FOXO3 under hypoxia relies on AKT1-mediated phosphorylation. Considering that the phosphorylation of 14-3-3 upon hypoxic exposure could also prevent the binding of FOXO3 to 14-3-3, thus promoting the nuclear translocation of FOXO3, it is plausible that JNK1/2 and AKT1 jointly participate in regulating the nuclear translocation of FOXO3 under hypoxia. Additional investigations are necessary to thoroughly elucidate the distinct mechanisms governing the nuclear-cytoplasmic shuttling of FOXO1 and FOXO3 in response to hypoxia.

Follicular atresia is intimately associated with the processes of both apoptosis and autophagy in ovarian GCs 34. The hypoxic microenvironment within ovarian follicles has emerged as a pivotal intrinsic factor capable of triggering follicular atresia, concurrently leading to increased levels of autophagy and apoptosis in GCs 1. However, the interplay between autophagy and apoptosis in the context of hypoxic GCs remains elusive. In this study, we demonstrated that the FOXO1 transcription factor promotes apoptosis in GCs through FOXO3-dependent autophagy upon hypoxia exposure. By providing a profound understanding of the regulatory pathways controlling GC apoptosis under hypoxic conditions, our insights offer fresh perspectives in comprehending the mechanisms underlying follicular atresia. In summary, our findings offer a comprehensive understanding of how FOXO1 coordinates the regulation of FOXO3 to modulate autophagic death in the challenging context of hypoxia.

## Supplementary Material

Supplementary figures and tables.
