# Supplementary material for: FOXO1-mediated nuclear sequestration of STAT3 and AKT1 triggers FOXO3-dependent autophagic death in hypoxic granulosa cells
Source: Int J Biol Sci. 2024 Nov 4;20(15):5939–58. doi: 10.7150/ijbs.101309 (PMC11628327; doi:10.7150/ijbs.101309)
Supplement: Supplementary file 1 — Supplementary figures and tables. [file ijbsv20p5939s1.pdf]

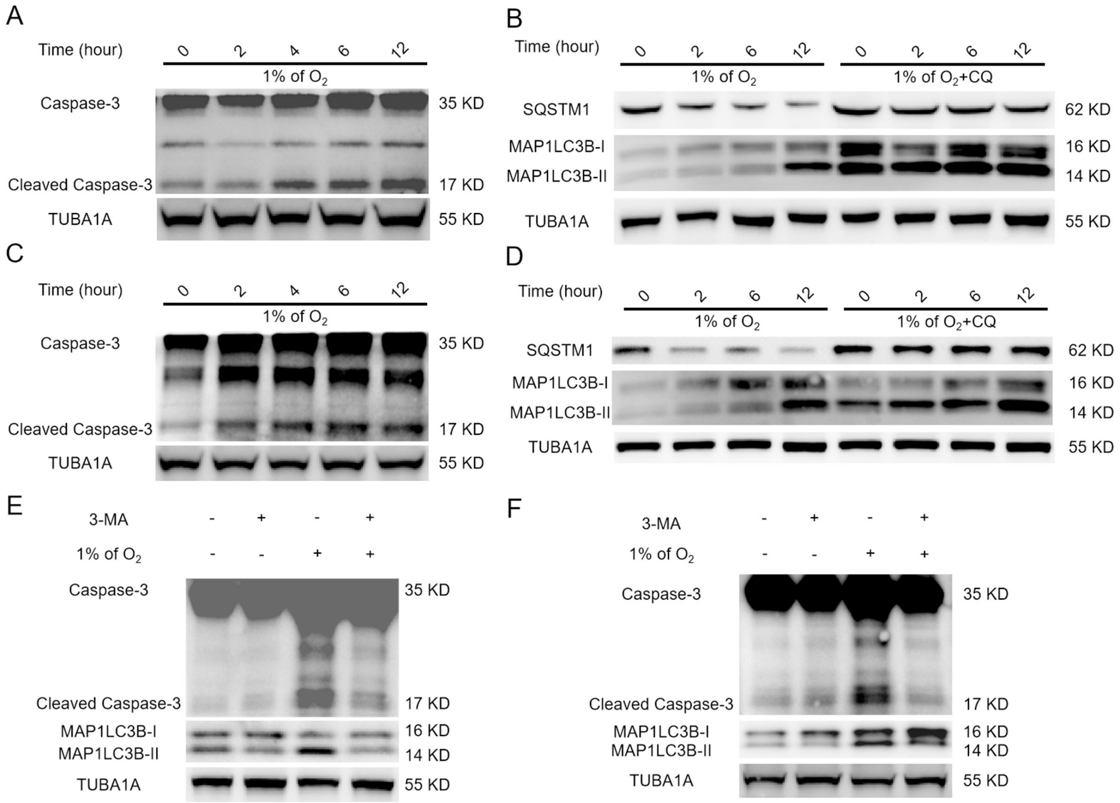

Figure S1. Hypoxia induces apoptosis by activating autophagy in NIH/3T3 and 293T cells. (A and C) NIH/3T3 (A) or 293T (C) cells were cultured under hypoxia (1% O_2) for 0, 2, 4, 6, or 12 h before the protein level of cleaved caspase-3 was determined by western blot. (B and D) NIH/3T3 (B) or 293T (D) cells were pre-treated with or without CQ (50 μM) and then cultured under hypoxia (1% O_2) for 0, 2, 4, 6, or 12 h before the protein level of SQSTM1 and MAP1LC3B were determined by western blot. (E and F) NIH/3T3 (E) or 293T (F) cells were cultured under normoxia (21% O_2) or hypoxia (1% O_2) for 12 h in the presence or absence of 3-MA (5 mM) before the protein level of cleaved caspase-3 was determined by western blot analysis.

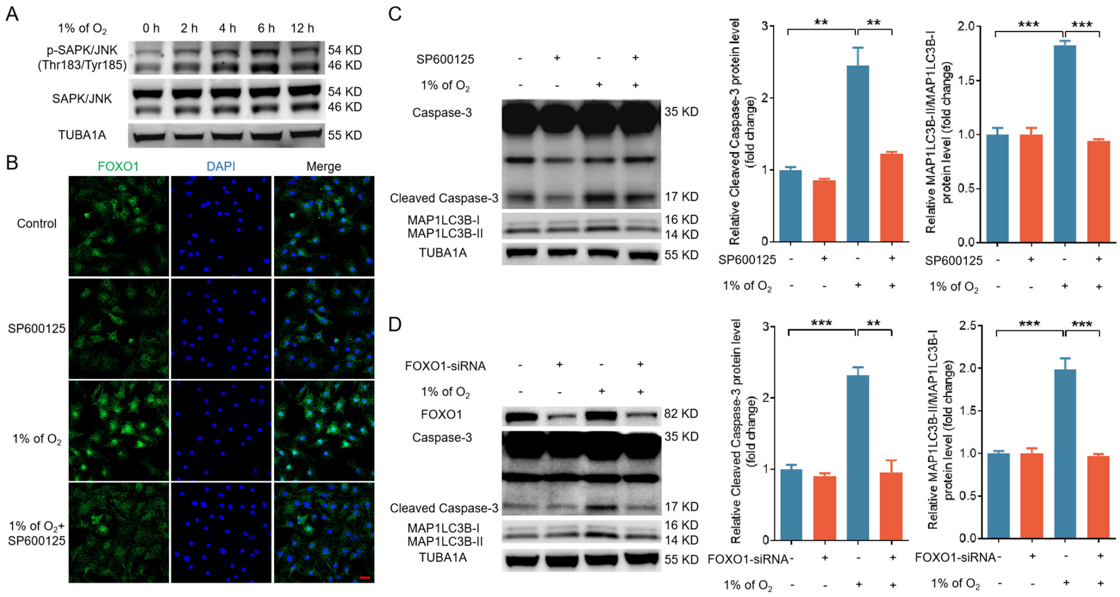

Figure S2. The JNK1/2-FOXO1 signaling pathway is implicated in the hypoxia-induced autophagic death of GCs. (A) Primary porcine GCs were cultured under hypoxia (1% O_2) for 0, 2, 4, 6, or 12 h before the protein levels of p-JNK1/2 (Thr183/Tyr185) and JNK1/2 were determined by western blot. (B and C) GCs were cultured under normoxia (21% O_2) or hypoxia (1% O_2) for 12 h in the presence or absence of JNK1/2 inhibitor SP600125 (10 μM). The subcellular localization of FOXO1 was observed by laser confocal-scanning microscopy (B), and the protein level of cleaved caspase-3 was determined by western blot (C). Bar, 50 μm. (D) GCs transfected with scramble control siRNA or siRNAs against *FOXO1* were cultured for another 12 h under normoxia (21% O_2) or hypoxia (1% of O_2). Protein levels of FOXO3 and cleaved caspase-3 were determined by western blot. The protein bands were quantified with densitometry using ImageJ 1.42q software.

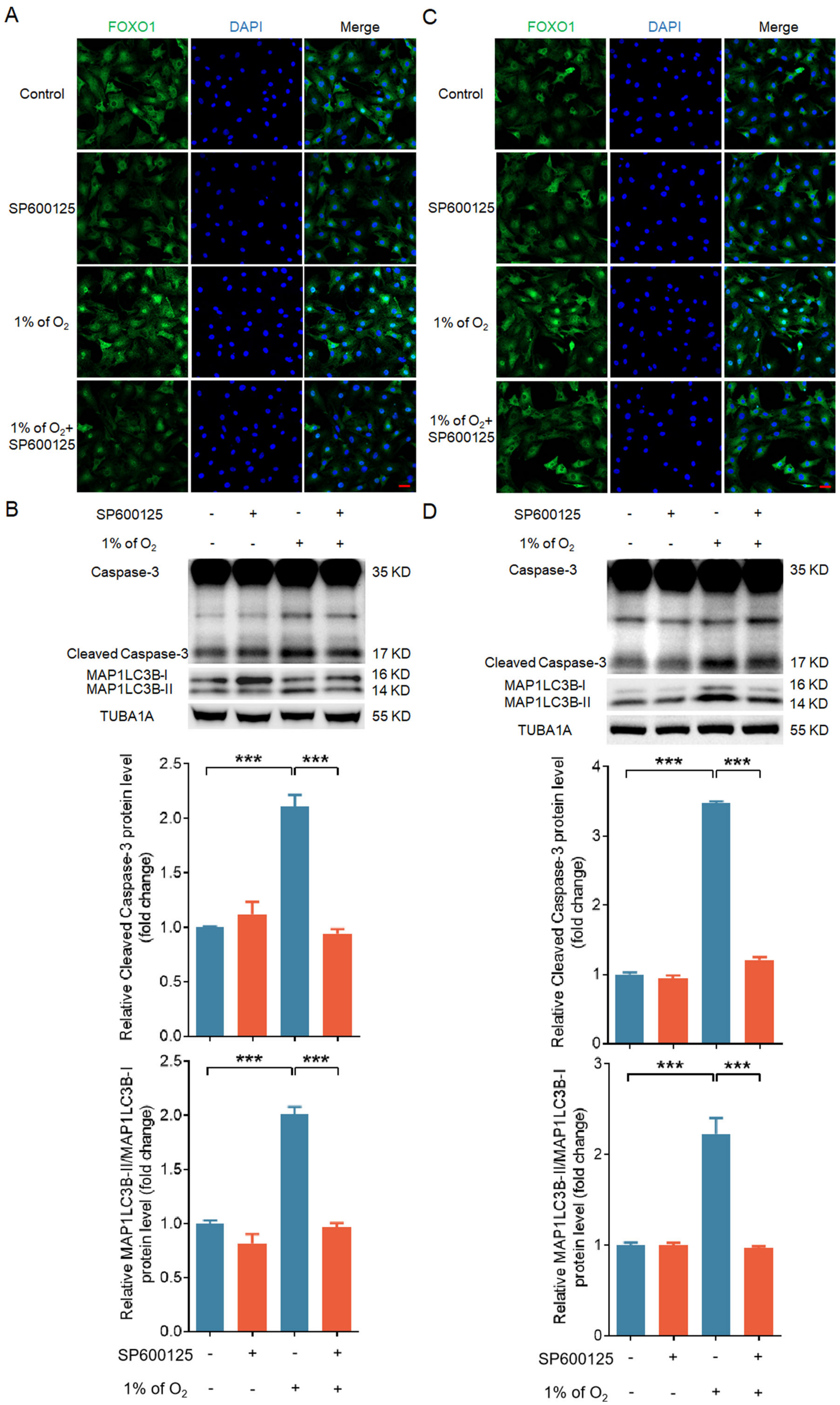
A
FOXO1
DAPI
Merge
Control
SP600125
1% of O_2
1% of O_2+ SP600125
B
SP600125 - + - +
1% of O_2 - - + +
Caspase-3
35 KD
Cleaved Caspase-3
17 KD
MAP1LC3B-I
MAP1LC3B-II
16 KD
14 KD
TUBA1A
55 KD
Relative Cleaved Caspase-3 protein level (fold change)
Relative MAP1LC3B-II/MAP1LC3B-I protein level (fold change)
***
C
FOXO1
DAPI
Merge
Control
SP600125
1% of O_2
1% of O_2+ SP600125
D
SP600125 - + - +
1% of O_2 - - + +
Caspase-3
35 KD
Cleaved Caspase-3
17 KD
MAP1LC3B-I
MAP1LC3B-II
16 KD
14 KD
TUBA1A
55 KD
Relative Cleaved Caspase-3 protein level (fold change)
Relative MAP1LC3B-II/MAP1LC3B-I protein level (fold change)
***

Figure S3. JNK1/2 is implicated in hypoxia-induced autophagic death of NIH/3T3 or 293T cells. (A–D) NIH/3T3 or 293T were cultured under normoxia (21% O_2) or hypoxia (1% of O_2) for 12 h in the presence or absence of JNK1/2 inhibitor SP600125 (10 μM). The subcellular localization of FOXO1 was observed by laser confocal-scanning microscopy (A and C), and the protein level of cleaved caspase-3 was determined by western blot (B and D). Bar, 50 μm. The protein bands were quantified with densitometry using ImageJ 1.42q software.

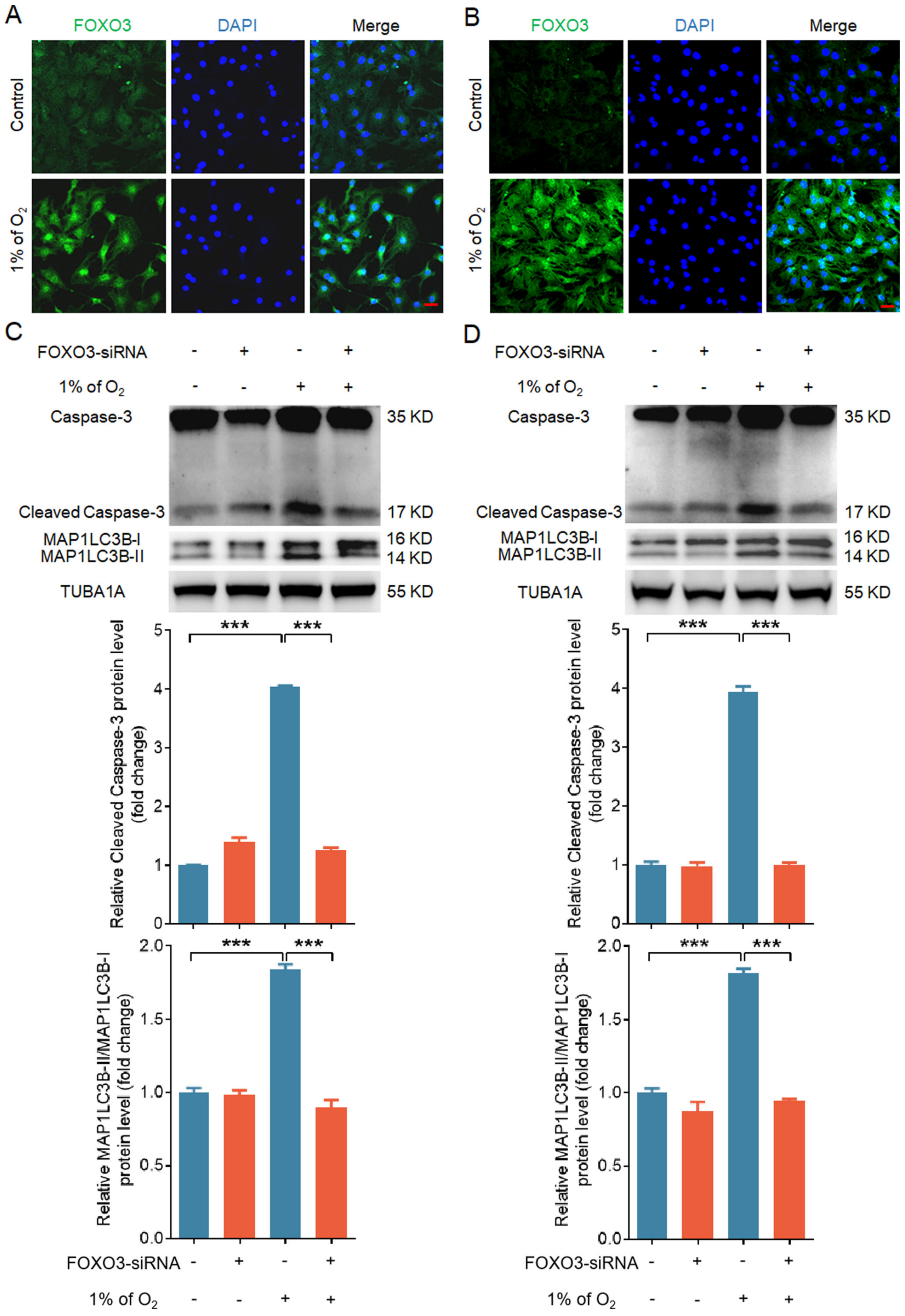

Figure S4. Hypoxia induces autophagic death of NIH/3T3 and 293T cells by promoting the expression and nuclear import of FOXO3. (A and B) NIH/3T3 (A) or 293T (B) cells cultured under normoxia (21% O_2) or hypoxia (1% O_2) for 6 h were

collected to observe the subcellular localization of FOXO3 by immunofluorescence assay. The scale bar represents 50 μm. (C and D) NIH/3T3 (C) or 293T (D) cells transfected with FOXO3-siRNA or scramble control siRNA for 12 h were cultured for another 12 h under normoxia (21% O_2) or hypoxia (1% O_2), and the proteins levels of MAP1LC3B or cleaved caspase-3 were determined by western blot. The protein bands were quantified with densitometry using ImageJ 1.42q software.

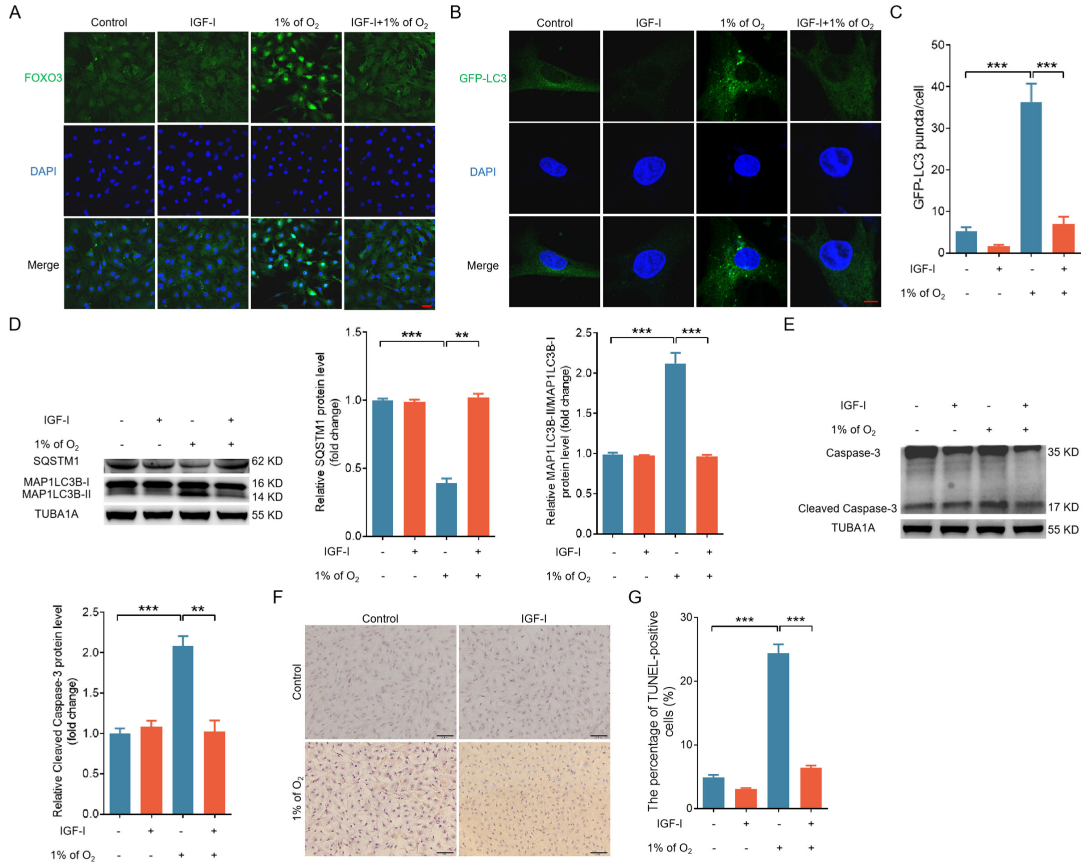

Figure S5. IGF-I prevents FOXO3-mediated autophagy/apoptosis in hypoxic GCs. (A–G) Primary porcine GCs treated with 10 nM of IGF-I were cultured under normoxia (21% O_2) or hypoxia (1% O_2) for 12 or 24 h. Immunofluorescent staining was performed to observe the subcellular localization of FOXO3. (A) The formation of autophagic puncta was examined by laser confocal-scanning microscopy (B). Bar, 5 µm. The quantification of GFP-LC3B puncta per cell is shown in (C). The protein levels of SQSTM1, MAP1LC3B (D), and cleaved caspase-3 (E) were measured by western blot analysis. The protein bands were quantified with densitometry using ImageJ 1.42q software. The apoptosis rate of GCs was determined using TUNEL staining (F), and the percentage of TUNEL-positive cells was quantified (G).

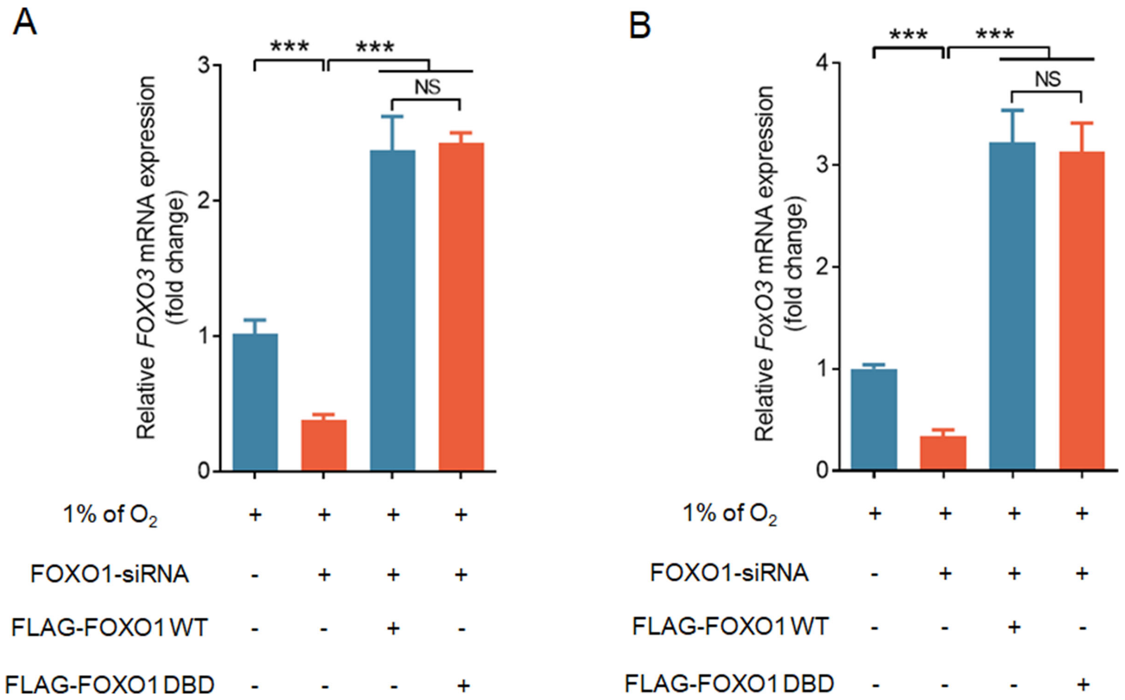

Figure S6. FOXO1 promotes FOXO3 expression without direct DNA binding in hypoxic NIH/3T3 and 293T cells. (A and B) After treatment with FOXO1-siRNA for 12 h, NIH/3T3 and 293T cells were transfected with FLAG-tagged FOXO1-expressing vectors, including FOXO1-WT (WT) and FOXO1-DBD/FOXO1$^{N208A,\ H212R}$ and then cultured under hypoxia (1% O_2) for another 12 h. The mRNA level of FOXO3 was measured by qRT-PCR.

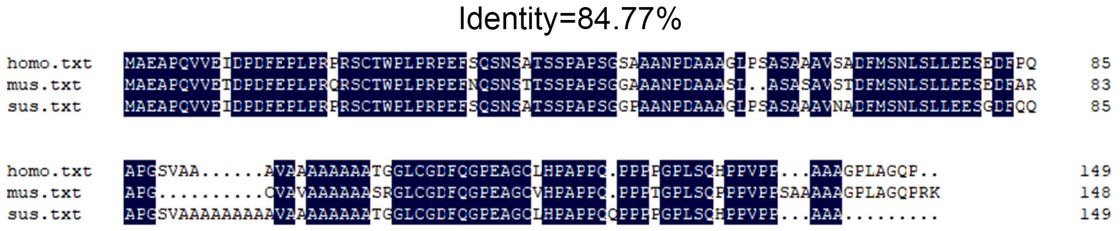

Figure S7. Sequence alignment of the STAT3 binding domain within FOXO1 protein from human, porcine, and murine cells.

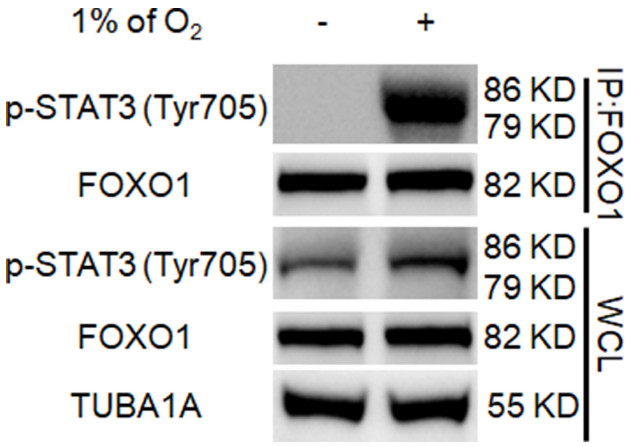

Figure S8. Tyr705 phosphorylation of STAT3 is not an essential element in the interaction between STAT3 and FOXO1. Primary porcine GCs were cultured under normoxia (21% O_2) or hypoxia (1% O_2) for 12 h. Cell lysates were subsequently collected for the evaluation of FOXO1 and p-STAT3 (Tyr705) expression through immunoblotting and investigation of the interaction between FOXO1 and p-STAT3 (Tyr705) through IP.

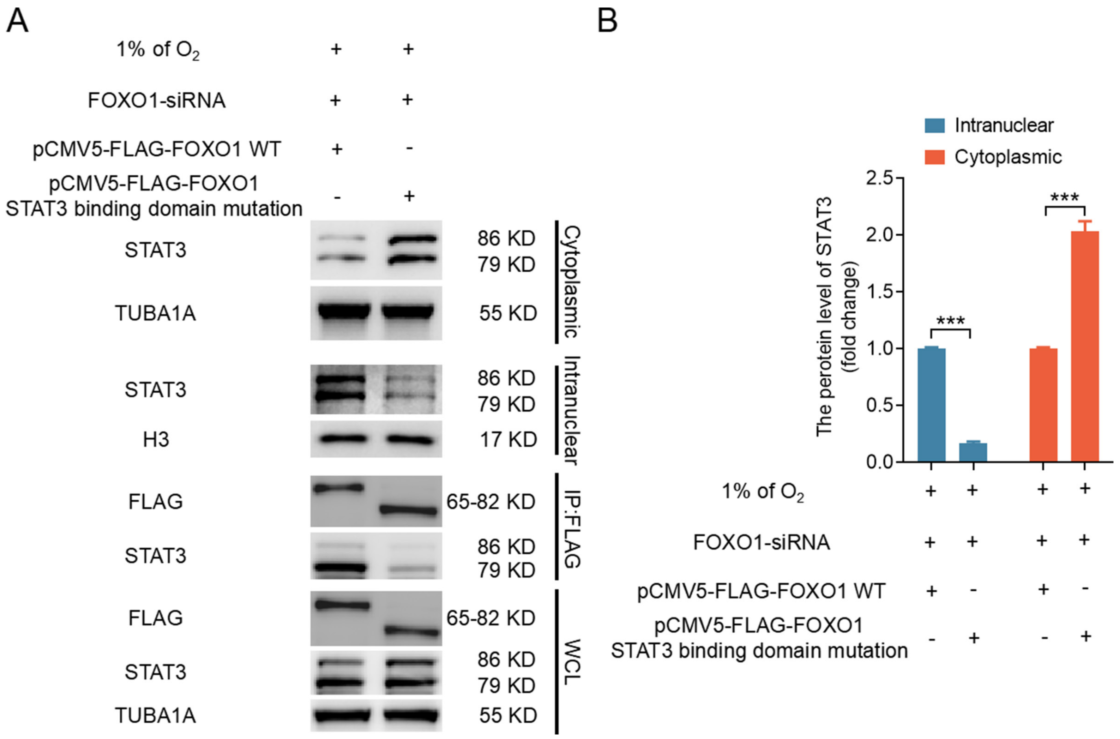

Figure S9. The truncated FOXO1 lacking the STAT3-binding domain disrupts FOXO1-STAT3 interaction and inhibits STAT3 nuclear translocation. (A and B) Primary porcine GCs were cultured under hypoxic conditions (1% O_2) and treated with FOXO1-siRNA for 12 h, followed by transfection with plasmids encoding either wild-type FOXO1 (FOXO1-WT) or a FOXO1 mutant lacking the STAT3-binding domain (FOXO1-ΔSTAT3). The cells were subsequently cultured under hypoxia (1% O_2) for another 12 h, and the interaction between FOXO1 and STAT3 was examined by IP (A). The cytoplasmic and nuclear fractions were subjected to western blot analysis to assess the expression of STAT3 (A). The protein levels were then measured using densitometry with ImageJ software (B).

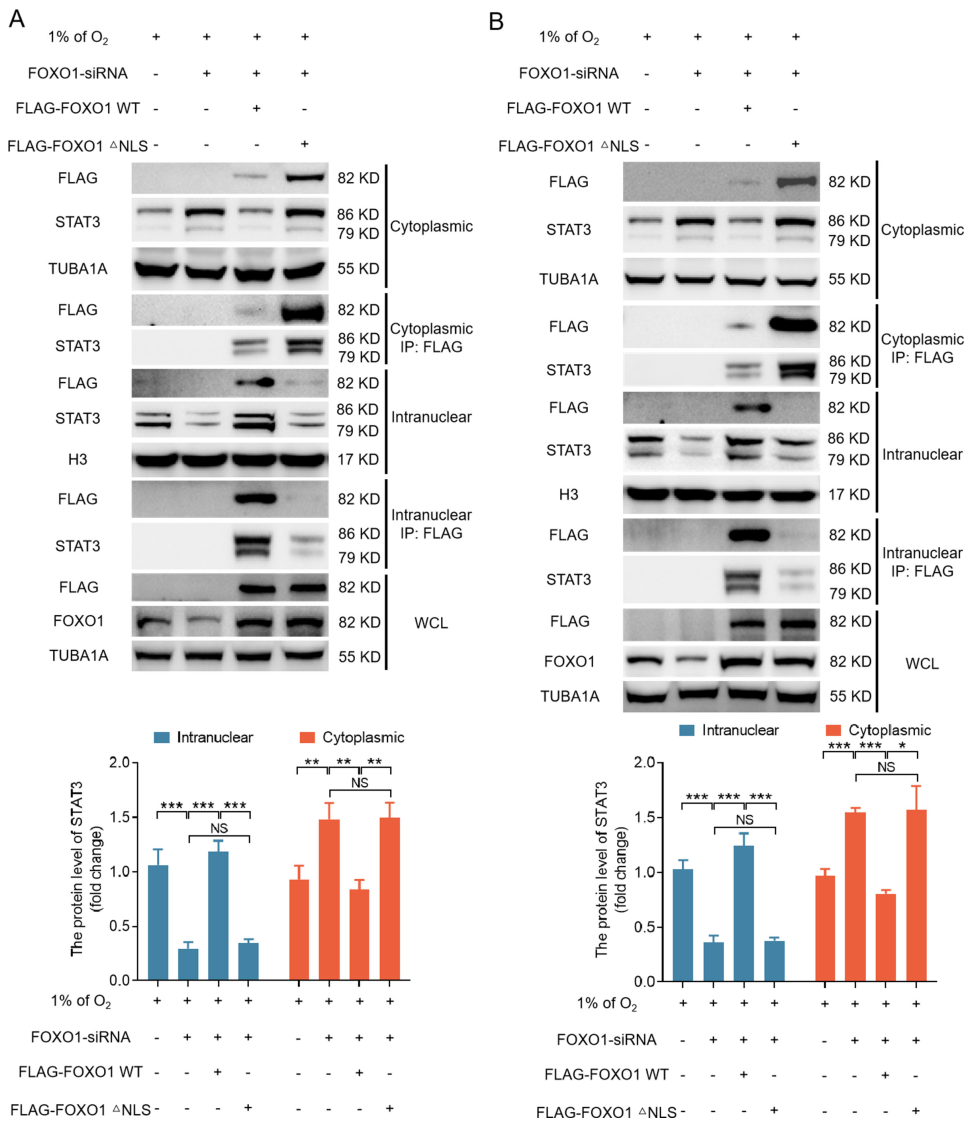

Figure S10. Hypoxia-induced nuclear translocation of FOXO1 facilitates the nuclear entry of STAT3 in NIH/3T3 and 293T cells. (A and B) After treatment with FOXO1-siRNA for 12 h, FLAG-tagged FOXO1-expressing vectors, including FOXO1-WT and FOXO1-△NLS, were transfected into NIH/3T3 (A) or 293T (B) cells, which were then cultured under hypoxia (1% O_2) for 12 h. The interaction between FOXO1 and STAT3 in the nucleus or cytoplasm was examined by IP. The protein bands were quantified with densitometry using ImageJ 1.42q software.

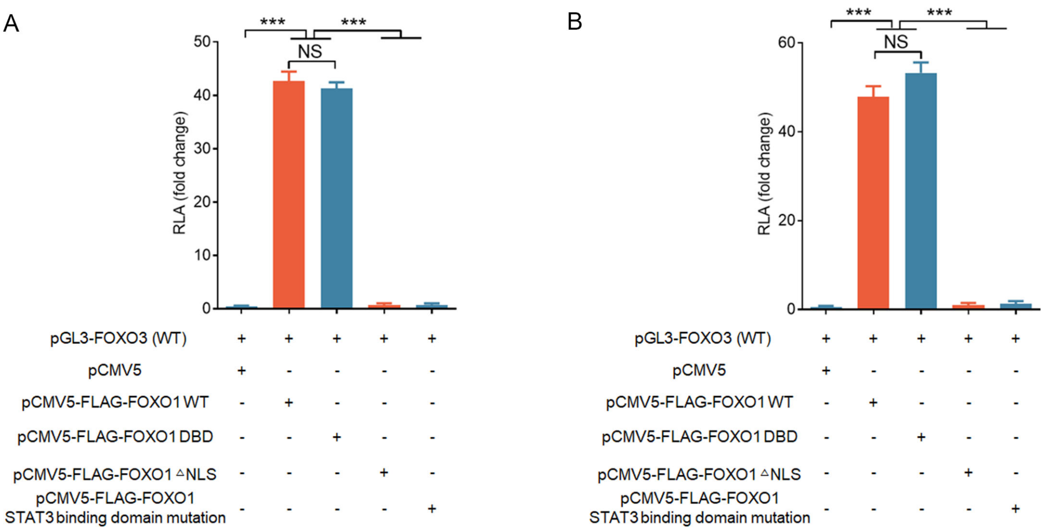

Figure S11. FOXO1-mediated nuclear transport of STAT3 activates the transcriptional expression of FOXO3 in NIH/3T3 or 293T cells. (A and B) FOXO3 reporter activities in NIH/3T3 (A) or 293T (B) cells co-transfected with FOXO1 expression vectors (FOXO1-WT, FOXO1-DBD, FOXO1-△NLS, or the STAT3 binding domain mutant) or STAT3-WT and FOXO3 promoter constructs for 24 h are shown. The reporter activities were normalized to those of pRL-TK.

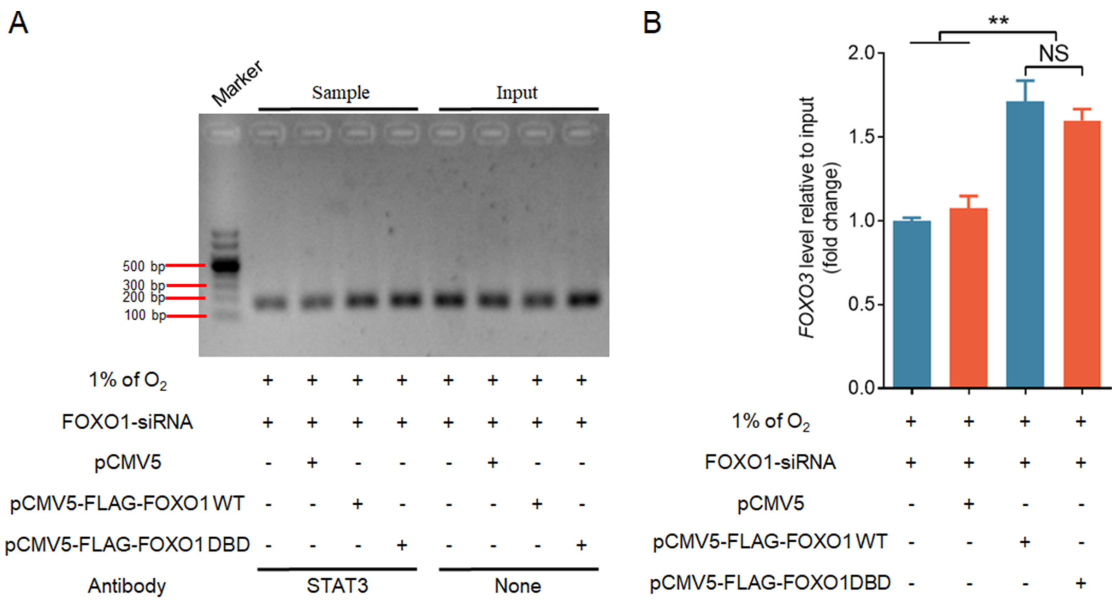

Figure S12. Overexpression of both FOXO1-WT and FOXO1-DBD enhances the binding of STAT3 to *FOXO3* promoter under hypoxia. (A and B) After treatment with FOXO1-siRNA for 12 h, FLAG-tagged FOXO1-WT or FOXO1-DBD expression vectors were transfected into primary porcine GCs. The cells were then cultured under hypoxia (1% O_2) for 12 h, and DNA was isolated from the precipitated complexes as a template for qRT-PCR. The qRT-PCR products were analyzed on a 2% agarose gel (A) and quantified with densitometry using ImageJ 1.42q software (B).

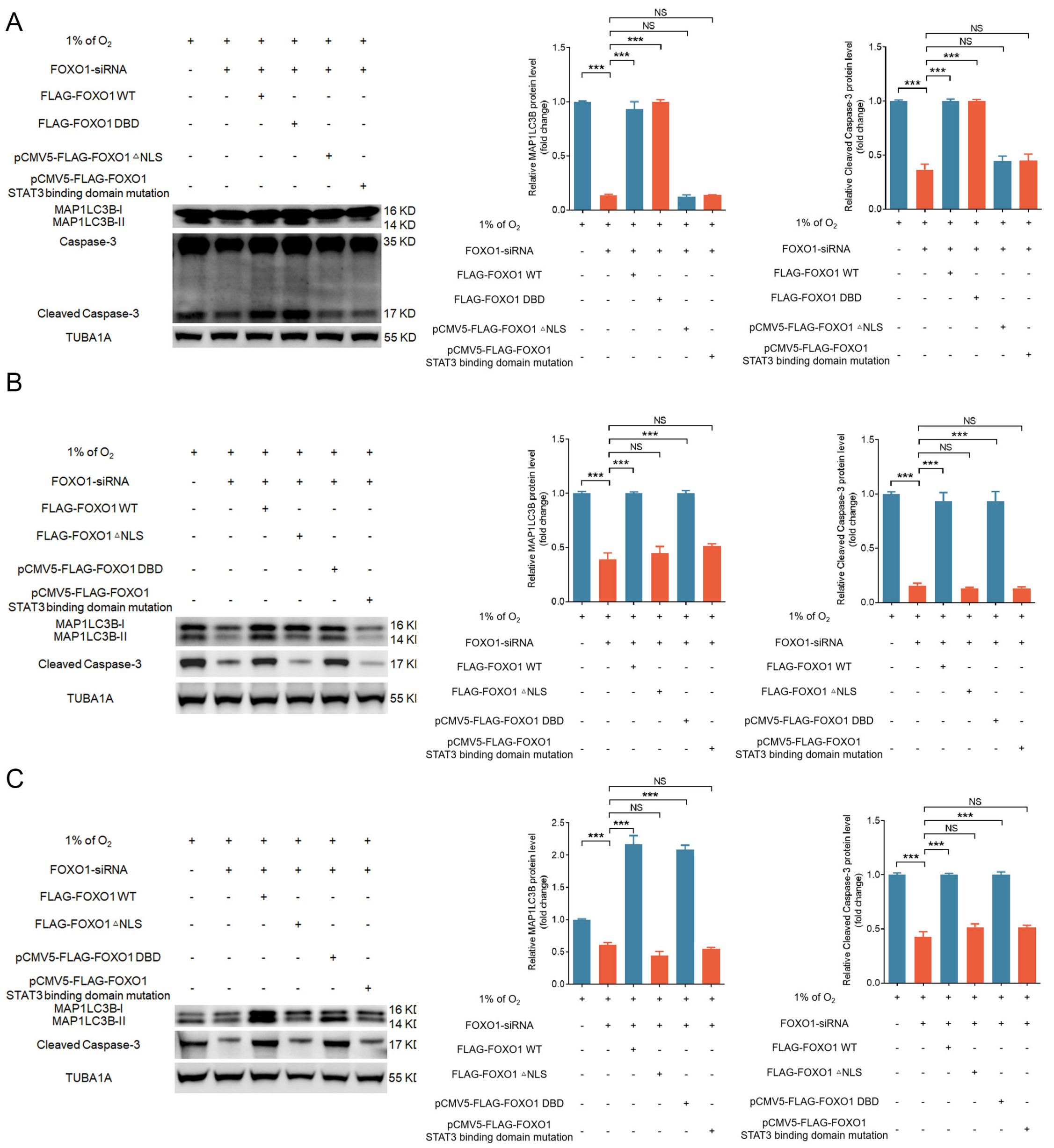

Figure S13. FOXO1 promotes autophagy and apoptosis by binding to STAT3 and translocating STAT3 into the nucleus. (A–C) Primary porcine GCs (A), 293T cells (B), or NIH/3T3 cells (C) cultured under hypoxia (1% O2) were treated with FOXO1-siRNA for 12 h and then transfected with FLAG-tagged FOXO1-expressing vectors, including FOXO1-WT, FOXO1-DBD, FOXO1-△NLS, or the STAT3 binding domain mutant. The cells were then cultured under hypoxia (1% O2) for another 12 h, and the protein levels of cleaved caspase-3 and MAP1LC3B were

determined by western blot. The protein bands were quantified with densitometry using ImageJ 1.42q software.

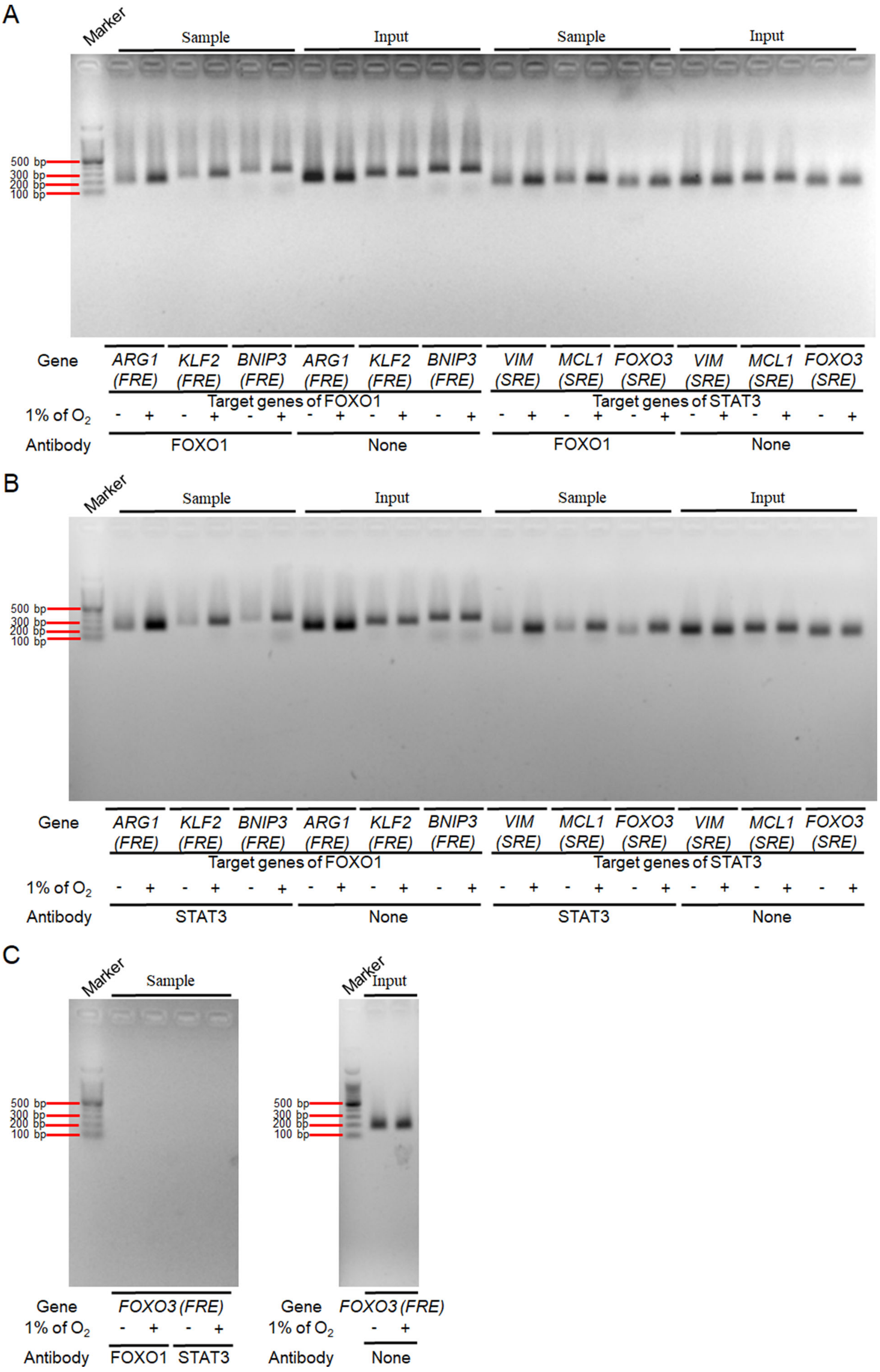

Figure S14. FOXO3 and STAT3 interact and bind together to the promoter of

downstream genes. (A) ChIP assays were performed using the FOXO1 antibody to investigate the binding of FOXO1 to the promoters of STAT3/FOXO1 target genes in primary porcine GCs following normoxia (21% O_2) or hypoxia (1% O_2) treatment for 12 h. DNA was isolated from the precipitated complexes as a template for qRT-PCR before the qRT-PCR products were analyzed on a 2% agarose gel. (B) ChIP assays were performed using the STAT3 antibody to assess the binding of STAT3 to the promoters of STAT3/FOXO1 target genes in GCs following normoxia (21% O_2) or hypoxia (1% O_2) treatment for 12 h. DNA isolated from the precipitated complexes served as a template for qRT-PCR, with the qRT-PCR products subsequently analyzed on a 2% agarose gel. (C) ChIP assays were performed using the antibodies against STAT3 or FOXO1, and qRT-PCR was performed with primers amplifying the *FOXO3* promoter containing the FRE motif. The amplicons were then analyzed on a 2% agarose gel.

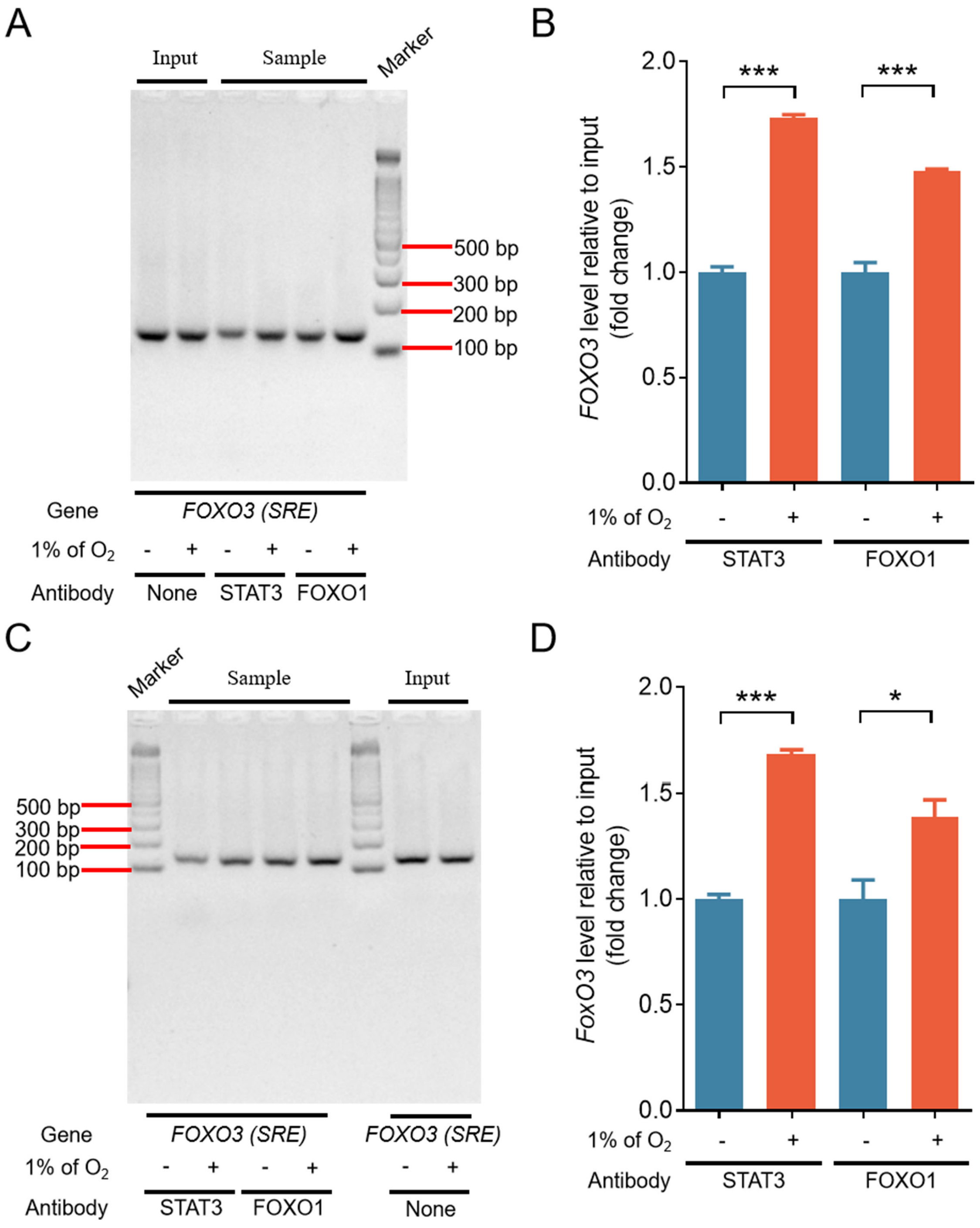

Figure S15. FOXO3 and STAT3 interact and bind together to the promoter of FOXO3 in NIH/3T3 and 293T cells. (A–D) ChIP assays were conducted using the FOXO1 or STAT3 antibody to investigate the binding of FOXO1/STAT3 to the promoter of FOXO3 target genes in NIH/3T3 (A and C) or 293T (B and D) cells following normoxia (21% O_2) or hypoxia (1% O_2) treatment for 12 h. DNA was isolated from

the precipitated complexes as a template for qRT-PCR. The qRT-PCR products were then analyzed on a 2% agarose gel (A and C) and quantified with densitometry using ImageJ 1.42q software (B and D).

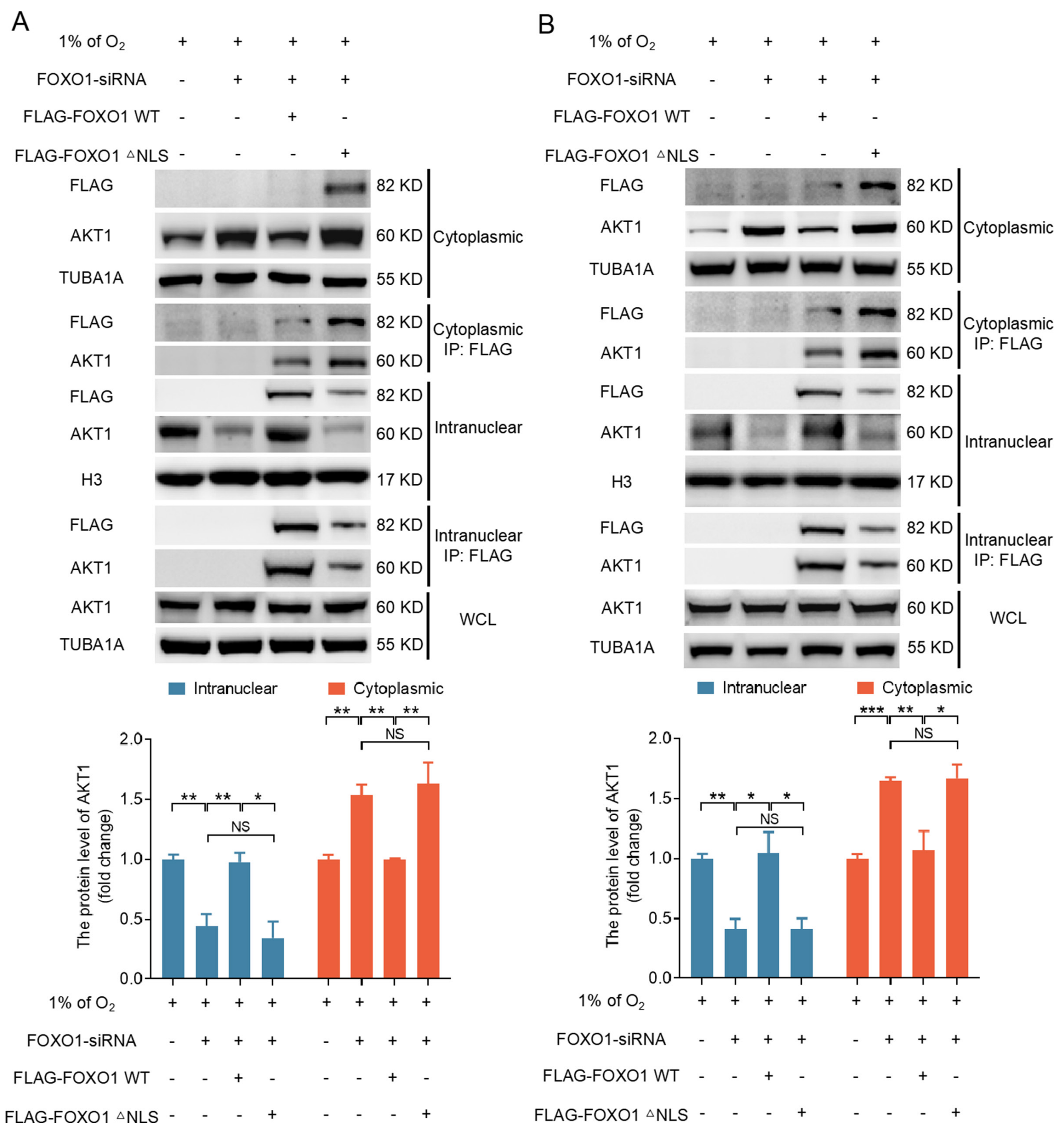

Figure S16. FOXO1 forms a complex with AKT1 and facilitates AKT1 nuclear translocation in hypoxic NIH/3T3 and 293T cells. (A and B) After treatment with FOXO1-siRNA for 12 h, FLAG-tagged FOXO1-expressing vectors, including FOXO1-WT and nuclear location signal mutant FOXO1 (△NLS) were transfected into NIH/3T3 (A) or 293T (B) cells, which were then cultured under hypoxia (1% O_2) for 12 h. The interaction between FOXO1 and AKT1 in the nucleus or cytoplasm was examined by IP, and the protein bands were quantified with densitometry using ImageJ 1.42q software.

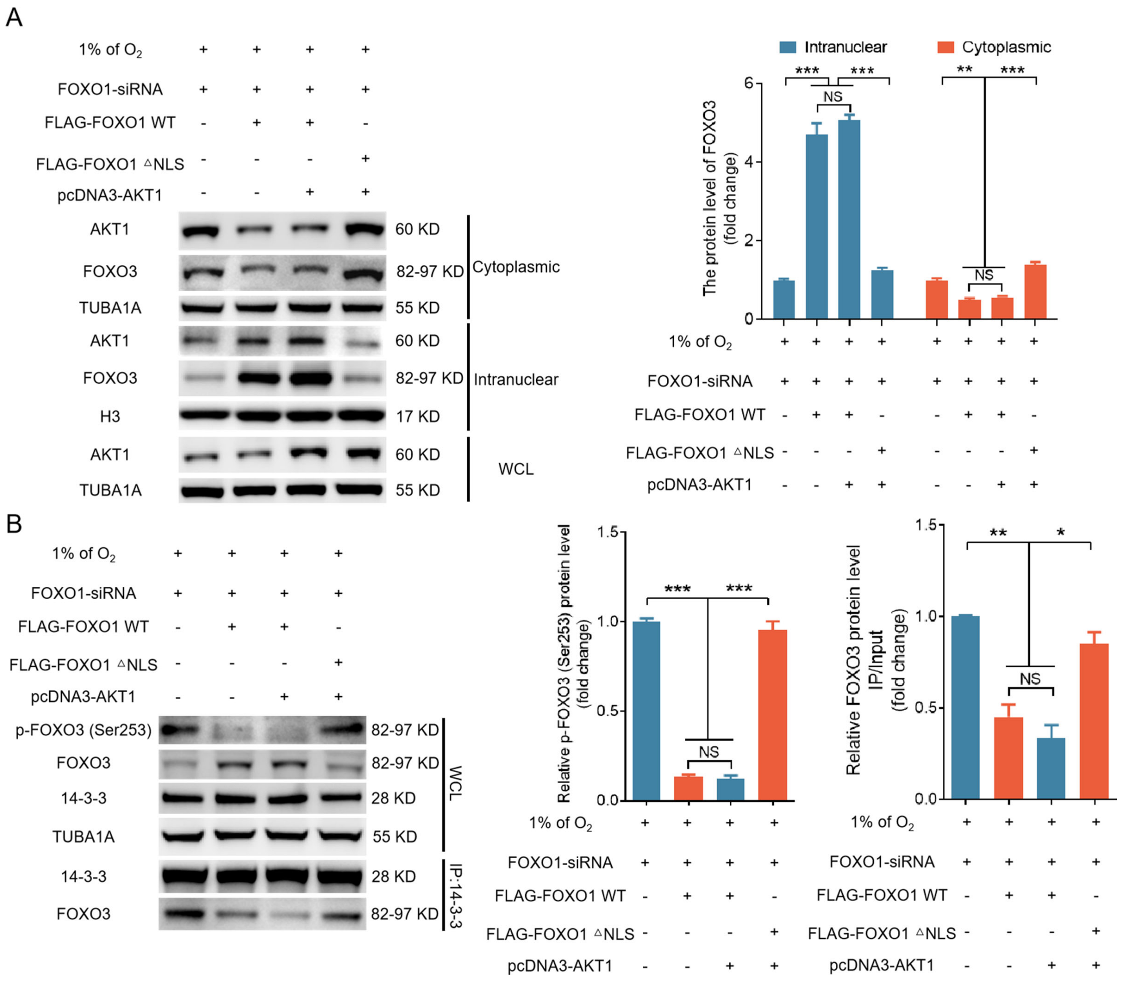

Figure S17. FOXO1-mediated AKT1 nuclear localization induces nuclear transportation of FOXO3 in hypoxic NIH/3T3 cells. (A and B) After treatment with FOXO1-siRNA for 12 h, GCs were co-transfected with FLAG-tagged FOXO1-WT or FOXO1-△NLS with or without AKT1 expression vector and then cultured under hypoxia (1% O_2) for 12 h. The protein levels of FOXO3 and AKT1 in the nucleus or cytoplasm were examined by western blot (A). The expression of p-FOXO3 (Ser253) and the interaction between FOXO3 and 14-3-3 were determined by immunoblotting and IP, respectively (B). The protein bands were quantified with densitometry using ImageJ 1.42q software.

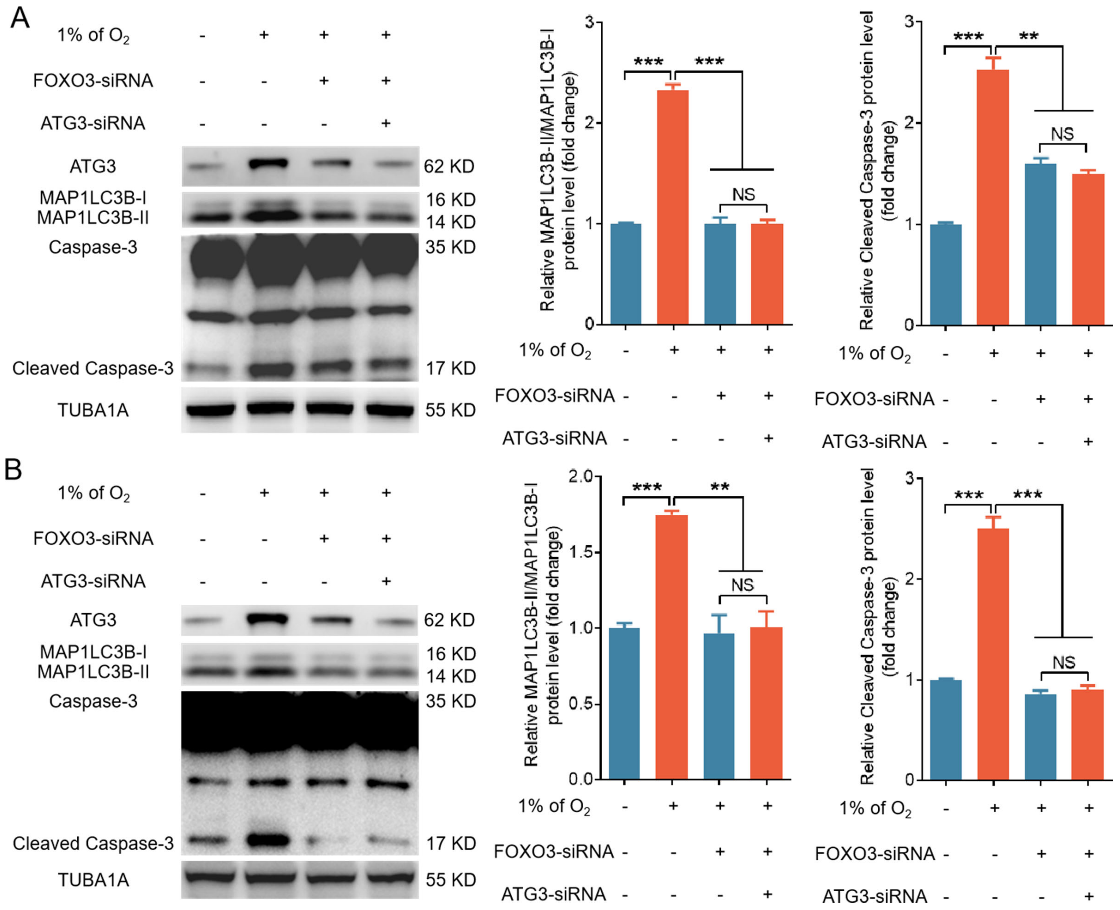

Figure S18. FOXO3 acts through ATG3 to promote autophagy and autophagy-dependent apoptosis in hypoxic NIH/3T3 and 293T cells. (A and B) NIH/3T3 (A) or 293T (B) cells transfected with FOXO3-siRNA and/or ATG3-siRNA were cultured under normoxia (21% O_2) or hypoxia (1% O_2) for 12 h. The protein levels of MAP1LC3B and cleaved caspase-3 were determined by western blot. The protein bands were quantified with densitometry using ImageJ 1.42q software.

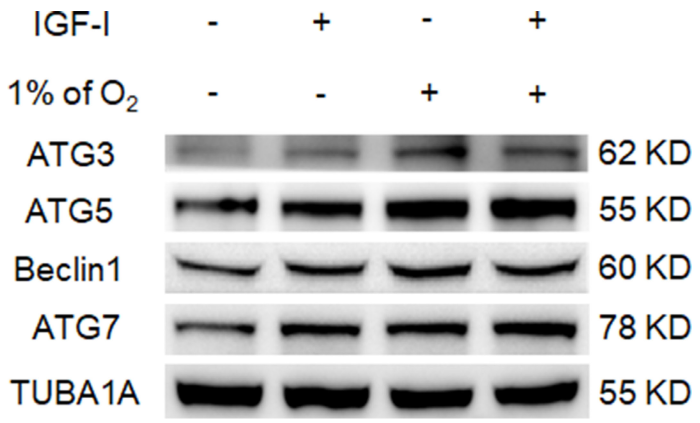

Figure S19. IGF-I inhibits ATG3 expression under hypoxia. Primary porcine GCs treated with 10 nM of IGF-I were cultured under normoxia (21% O_2) or hypoxia (1% O_2) for 12 h. Immunoblotting was then performed to examine the expression of autophagy-related proteins, including ATG3, ATG5, Beclin1, and ATG7.

Supplementary Table S1 siRNA sequences.

siRNA Name	Sense（5'-3'）	Antisense（5'-3'）
Scrambled siRNA (Sus scrofa)	UUCUCCGAACGUGUC ACGUTT	ACGUGACACGUUCGGAG AATT
FOXO3-siRNA (*Sus scrofa*)	GGAGCUUGGAAUGUG ACAUTT	AUGUCACAUUCCAAGCU CCTT
FOXO1-siRNA (*Sus scrofa*)	GCAUGUUCAUUGAGCGC UUTT	AAGCGCUCAAUGAACAUG CTT
ATG3-siRNA1 (*Sus scrofa*)	GGUGCAAACAGAUGG AAUATT	UAUUCCAUCUGUUUGCA CCTT
ATG3-siRNA2 (*Sus scrofa*)	GCUGCAGAUAUGGAA GAAUTT	AUUCUUCCAUAUCUGCA GCTT
ATG3-siRNA3 (*Sus scrofa*)	GAGGUGAUGAAGAAG AUUATT	UAAUCUUCUUCAUCACC UCTT
Scrambled siRNA (*Homo sapiens*)	CAAUGCUACUAAGUC CCUUCUUACA	UGUAAGAAGGGACUUA GUAGCAUUG
FOXO3-siRNA (*Homo sapiens*)	UCAGAAAGGAGCAAG UGGAGGUGGA	UCCACCUCCACUUGCUC CUUUCUGA
FOXO1-siRNA (*Homo sapiens*)	CAAUUCGUCAUAAUC UGUCCCUACA	UGUAGGGACAGAUUAU GACGAAUUG

ATG3-siRNA (*Homo sapiens*)	GAAAGGCACUGGAAG UGGCUGAGUA	UACUCAGCCACUUCCAG UGCCUUUC
Scrambled siRNA (*Mus musculus*)	GGGAGUAGUAAGAGU AAGGCAAAUA	UAUUUGCCUUACUCUUA CUACUCCC
FoxO3-siRNA (*Mus musculus*)	GGCAAGAGCUCUUGG UGGAUCAUCA	UGAUGAUCCACCAAGAG CUCUUGCC
FoxO1-siRNA (*Mus musculus*)	CAGAAUGAAGGAACU GGAAAGAGUU	AACUCUUUCCAGUUCCU UCAUUCUG
Atg3-siRNA (*Mus musculus*)	GGGAAGAAUUGAAAG UGAAGGCAUA	UAUGCCUUCACUUUCAA UUCUUCCC

Supplementary Table S2 Primer sequences for RT-qPCR.

Gene Name	Primer Sequence (5'→3')
TUBA1A (*Sus scrofa*)	F: AAGAGTCGCGCTGTAAGAAG<br>R: AATGACTGTGGGTTCCAGGTC
FOXO1 (*Sus scrofa*)	F: AAGACCGCTTTACAAGTGCC<br>R: TCAATGAACATGCCATCCAA
FOXO3 (*Sus scrofa*)	F: GCCGGCTGGAAGAACTCTAT<br>R: GCGGCTCTTGGTGTACTTGT
FOXO4 (*Sus scrofa*)	F: TCATCAGCCAGGCCATTGAA<br>R: TGTGGCGGATCGAGTTCTTC
FOXO3 (*Homo sapiens*)	F: GTCCGCGATCCTGTACGTG<br>R: CGTCTTCATCGTCCTCCTCC
FoxO3 (*Mus musculus*)	F: CTGGGGGAACCTGTCCTATG<br>R: TCATTCTGAACGCGCATGAAG
ATG3 (*Sus scrofa*)	F: CGTTTTCTGACTCCCGATCCC<br>R: CCAGCTGCCACAAACTCTTCT

Supplementary Table S3 Primer sequences for ChIP-qPCR.

Gene Name	Primer Sequence (5'→3')
FOXO3 (SRE) (*Sus scrofa*)	F: CAGGGAAGAAATGAATGACC<br>R: GTGATTGTTCCCTTTTAGCC
ARG1 (*Sus scrofa*)	F: TGCCAATTCCCAGCTTATCCA<br>R: AGGGTATAGAGCCAACCTCC
KLF2 (*Sus scrofa*)	F: TTCCCTCTAGCCTGTGGCTT<br>R: GGCCGTGTTGAGGATCAGTA
BNIP3 (*Sus scrofa*)	F: GCAGAGTCGTGGTGTCTGTAA<br>R: CGCTCCTTCTCCTCTCAGGAT

VIM (*Sus scrofa*)	F: GATACAGCTTGGGGACAGGTT<br>R: CCTCAGGTCTGTGGGTGACT
MCL1 (*Sus scrofa*)	F: ATGCACTTCCTTCTACAGCC<br>R: AGGTAGCTTTTTCTGCTCTGCT
FOXO3 (FRE) (*Sus scrofa*)	F: CTGTGGCTTAGGCACTCACC<br>R: GCATGCTTTGGATCCCGCTA
FOXO3 (SRE) (*Homo sapiens*)	F: GCTTCTCCTTCGCCGAGGT<br>R: ATCCGGAGTCACCGGGAAG
FoxO3 (SRE) (*Mus musculus*)	F: AAGGGATTGTGAAGGTGCGA<br>R: ATAGCTAACTGGAAGCGGGC
ATG3 (SRE) (*Sus scrofa*)	F: GGCTTTGCAGGGATCTAGGG<br>R: CCCGTGTACCATTCCTACCG

Supplementary Table S4 Primer sequences for *pGL3-FOXO3* vector construction.

**Gene Name**	**ID**	**Primer Sequence (5'→3')**
pGL3-FOXO3	G0195325-5-A_1	TGCAGGTGCCAGAACATTTCTCTATCGATAGGTAC<br>CATGCTGGCATTCCTTCTCAATCAACAACCTAAC
	G0195325-5-C_18	TGGTGGCTTTACCAACAGTACCGGAATGCCAAGC<br>TTGTGTTTTTTTATCTTTTTAAAGAAAACTATTT
